# Supplementary material for: Professional stakeholders’ expectations for the future of community pharmacy practice in England: a qualitative study
Source: BMJ Open. 2023 Oct 16;13(10):e075069. doi: 10.1136/bmjopen-2023-075069 (PMC10582972; doi:10.1136/bmjopen-2023-075069)
Supplement: Supplementary data [file bmjopen-2023-075069supp001.pdf]

BMJ Open

Professional stakeholders' expectations for the future of community pharmacy practice in England: a qualitative study

Supplemental material

Evgenia Paloumpi[a]*, Piotr Ozieranski[b], Margaret C Watson[c] and Matthew D. Jones[a]

[a]Department of Life Sciences, University of Bath, Bath, England.

[b]Department of Social & Policy Sciences, University of Bath, Bath, England.

[c]Strathclyde Institute of Pharmacy and Biomedical Sciences, University of Strathclyde, Glasgow, Scotland.

*The views expressed in this paper are purely those of the author. They do not necessarily reflect the views or official positions of the European Commission and the ERC Executive Agency.

Table S1: Consolidated criteria for reporting qualitative research (COREQ) 32-item checklist

	**Guide questions/description**	**Comment**	**Location in the manuscript (section)**
**Domain 1: Research team and reflexivity**			
Personal Characteristics			
1. Interviewer/ facilitator	Which author/s conducted the interview or focus group?	Evgenia Paloumpi (EP) conducted the interviews.	2.2
2. Credentials	What were the researcher's credentials? E.g. PhD, MD	EP had MPharm and MRes degrees. MW, PO and MJ each had a PhD. EP, MW and MJ were all registered pharmacists.	N/A
3. Occupation	What was their occupation at the time of the study?	EP was a PhD student at Department of Pharmacy and Pharmacology, University of Bath.<br>MW, PO and MJ were EP's supervisors and full-time academics.	N/A
4. Gender	Was the researcher male or female?	EP and MW were female.<br>PO and MJ were male.	N/A
5. Experience and training	What experience or training did the researcher have?	EP had undertaken several courses related to advanced qualitative health research before the study was conducted. The other authors had extensive training and experience in qualitative health research.	N/A
Relationship with participants			
6. Relationship established	Was a relationship established prior to study commencement?	EP had previously met some participants at professional events such as conferences.	N/A
7. Participant knowledge of the interviewer	What did the participants know about the researcher? e.g. personal goals, reasons for doing the research	Potential participants were sent written information explaining the aims of the study and that it informed part of a PhD project.<br><br>Prior the interviews, EP introduced herself again as a PhD student and described the purpose of the project, before answering any questions that participants may have had.	2.1

8. Interviewer characteristics	What characteristics were reported about the interviewer/facilitator? e.g. Bias, assumptions, reasons and interests in the research topic	EP is a pharmacist with a particular interest in health policy.<br>MW is a pharmacist with a particular interest in community pharmacy research.<br>MJ is a pharmacist with experience of work as a community pharmacy locum and research interests in medicines safety and medicines information.<br>PO is a health policy researcher.	N/A
**Domain 2: Study design**			
Theoretical framework			
9. Methodological orientation and Theory	What methodological orientation was stated to underpin the study? e.g. grounded theory, discourse analysis, ethnography, phenomenology, content analysis	Underpinning ontology and epistemology were interpretivist. A reflexive thematic analysis using inductive approaches was applied. The Walt and Gilson policy framework was used to develop the topic guide.	2.3
Participant selection			
10. Sampling	How were participants selected? e.g. purposive, convenience, consecutive, snowball	Organisational representatives were selected using stakeholder mapping and snowballing. Community pharmacists were selected purposively.	2.1
11. Method of approach	How were participants approached? e.g. face-to-face, telephone, mail, email	Organisational representatives were contacted directly in writing and by telephone.<br><br>Community pharmacists were approached via advertisements in the Royal Pharmaceutical Society online community pharmacy forum, 48 local pharmaceutical committees, the researchers' networks and social media.<br><br>A study information sheet was emailed to these individuals who agreed to take part.	2.1
12. Sample size	How many participants were in the study?	Twenty-five	3.1

13. Nonparticipation	How many people refused to participate or dropped out? Reasons?	No interviewees were recruited from the following groups: public representatives; pharmacy and allied healthcare professionals (The Association of Independent Multiple Pharmacies, Boots Pharmacists Association, Royal College of General Practitioners, British Medical Association, Royal College of Nursing); commissioners and policymakers (Department of Health and Social Care, All-Party Pharmacy Group); and experts and collaborators (The King's Fund). The most common reasons for non-recruitment were either no response received or time restrictions of interviewees.	N/A
Setting			
14. Setting of data collection	Where was the data collected? e.g. home, clinic, workplace	Interviewees could determine whether they preferred to be interviewed face-to-face or by telephone. All face-to-face interviews took place in nonclinical professional area.<br><br>Five interviews were conducted in person at a location convenient to participants (e.g. participants' offices), 19 by telephone and one by Skype using videocall.	2.2
15. Presence of non-participants	Was anyone else present besides the participants and researchers?	No.	N/A
16. Description of sample	What are the important characteristics of the sample? e.g. demographic data, date	-	3.1 and Table S2 in this supplementary material
Data collection			
17. Interview guide	Were questions, prompts, guides provided by the authors? Was it pilot tested?	An interview topic guide (see below) was developed based on a recent policy review, previous research, supervisory team discussions and the Walt and Gilson policy framework (content, context, process and actors). The final interview guide included 11, broad, open-ended questions and probes to allow a flexible conversation with participants, with the aim of understanding their views on how	Below

		pharmaceutical care in the community would be provided in 2030. The topic guide and interviewer's technique were piloted interview with a stakeholder who was not included in the study as a participant. The pilot provided useful insights and feedback on interview conduct (e.g. ensuring availability for the interview duration before commencing it). The topic guide evolved progressively as interviews were carried out, to ensure that findings informed subsequent interviews. Questions 7, 8 and 9 were added to the topic guide as many participants referred to these topics during their interviews.	
18. Repeat interviews	Were repeat interviews carried out? If yes, how many?	No.	N/A
19. Audio/visual recording	Did the research use audio or visual recording to collect the data?	All interviews were audio-recorded (with permission) and later transcribed verbatim.	2.2
20. Field notes	Were field notes made during and/or after the interview or focus group?	Hand-written notes with key points and observations of the interview were also made by the researcher.	2.2
21. Duration	What was the duration of the interviews or focus group?	Interview duration varied from 35 to 75 minutes (mean = 60 minutes).	3.1
22. Data saturation	Was data saturation discussed?	Participants in this study were not recruited based on data saturation estimates as every stakeholder's view was considered useful for the future development of pharmaceutical care. Recruitment continued until all identified stakeholders had been interviewed or had declined.	2.1
23. Transcripts returned	Were transcripts returned to participants for comment and/or correction?	Transcripts were not returned to participants for comments due to time restrictions.	N/A
**Domain 3: Analysis and findings**			
Data analysis			

24. Number of data coders	How many data coders coded the data?	Coding was led by one researcher (EP), with regular discussion and agreement with the team.	2.3
25. Description of the coding tree	Did authors provide a description of the coding tree?		3.2-2.6
26. Derivation of themes	Were themes identified in advance or derived from the data?	This study used an inductive approach. The codes and the themes were informed by interview content.	2.3
27. Software	What software, if applicable, was used to manage the data?	NVivo12® software was used.	2.3
28. Participant checking	Did participants provide feedback on the findings?	No.	N/A
Reporting			
29. Quotations presented	Were participant quotations presented to illustrate the themes/findings? Was each quotation identified? e.g. participant number	Yes, quotations were presented and identified in a manner protecting participant confidentiality.	3.3-3.6
30. Data and findings consistent	Was there consistency between the data presented and the findings?	Yes, there was consistency between the data and the findings.	3.2-3.6
31. Clarity of major themes	Were major themes clearly presented in the findings?	Yes, major themes were clearly identified.	3.2-3.6
32. Clarity of minor themes	Is there a description of diverse cases or discussion of minor themes?	Yes, minor themes and diverse cases were clearly identified and related to major themes.	3.2-3.6

N/A; not applicable

Tong A, Sainsbury P, and Craig J. Consolidated criteria for reporting qualitative research (COREQ): a 32-item checklist for interviews and focus groups. *Int J Qual Health Care* 2007;19:349–57.

Table S2: Organisations with representatives invited to participate in this study

Type of stakeholder	Type of organisation	Organisations
External stakeholders	Public representatives	Healthwatch<br>National Voices<br>The Patients' Association
	Health professions	Royal College of General Practitioners (RCGP)<br>British Medical Association (BMA)<br>Royal College of Nursing (RCN)
	Commissioners and policymakers	Department of Health and Social Care (DHSC)<br>All Party Parliamentary Group on Pharmacy (APPG)<br>NHS England (NHSE)<br>NHS Clinical Commissioning (NHSCC)
	Experts and collaborators	UK universities<br>The King's Fund<br>Pharmacy media
Internal stakeholders	Pharmacy organisations	Royal Pharmaceutical Society (RPS)<br>Pharmaceutical Services Negotiating Committee (PSNC)<br>National Pharmacy Association (NPA)<br>Company Chemists' Association (CCA)<br>The Association of Independent Multiple Pharmacies (AIMp)<br>Pharmacists' Defence Association (PDA)<br>Boots Pharmacists' Association (BPA)<br>Association of Pharmacy Technicians UK (APTUK)

Interview topic guide

Opening

- Researcher introduction and brief overview explaining purpose of the study and describing the term "pharmaceutical care in the community" used for the study

  "*My name is Evina and I am a PhD student from University of Bath. As you already know, my research aims to develop a more evidence-based vision for the future of community pharmacy services in England. We are having this conversation to discuss your views on how pharmaceutical care will be provided in the community in the year 2030. And when I say pharmaceutical care, I would describe this discipline as helping patients use and manage medicines effectively, but also providing wider interventions that could create an impact on their quality of life*."

- Check participant has read Participant Information Sheet (provide a copy and time to read if they haven't)
- Ask participant if they have any questions about the study
- Explain to the participants about the informed consent
- Ask how much time the participant has available and plan questions – "*Our interview is not expected to last more than one hour but if you have other commitments, we can finish earlier than that.*"
- Secure consent
- Start recording the interview

Main Discussion

Probes will be used flexibly, in response to participant's answers.

1. How would you describe your [members'] role in relation to this sort of pharmaceutical care in the community?	**Role**:
2. In your view, in what ways might pharmaceutical care in the community be provided in the year 2030?<br><br>*Probe common topics from policy review:*<br>• *urgent and emergency care*<br>• *mental health (dementia friend)*<br>• *closer collaborations with primary care professionals*<br>• *long-term conditions*<br>• *patient-centred care*<br>• *new models of care*	**Ways**:
3. Which of these developments are more like likely to happen and why?	**More likely to happen & why**:

Probe common drivers from policy review:<br>• *unnecessary workload in general practice*<br>• *increased service demands*<br>• *funding allocation*<br>• *increased hospital admissions*	
4. We have already discussed the different ways you think that pharmaceutical care will be provided in the year 2030. Suppose that you had an opportunity to create the ideal model of pharmaceutical care in the community, but without increasing the total cost. What would this ideal model include?	**Ideal model:**
5. [If answer to Q4 different to Q3] Your ideal model is different to what you think will actually happen. Why do you think your ideal model is unlikely to come about?	**Why expectation different to ideal:**
6. What would make your ideal model more likely to come about?<br><br>*Probe common facilitators and barriers for Q5 and Q6:*<br>• *individual behaviour*<br>• *utilising technology*<br>• *supporting workforce*<br>• *providing evidence*<br>• *facilitate commissioning*	**What would make ideal more likely:**
7. What are the current barriers to pharmaceutical care provision in the community?	**Barriers:**
8. What are your views on the role of pharmacy technicians in pharmaceutical care?	**Technicians:**
9. What are your views on community pharmacy mergers and closures due to financial constraints?	**Mergers and closures:**
10. Do you feel there is anything else you would like to add?	**Additional:**

Closing

- Summarise main points of the discussion
- One last question from me “What other organisation or individual would you consider influential for the future of pharmaceutical care in the community?
- Ask if they would like a copy of the results to be sent to them
- Thank participants for their time

Table S3: Interviewee characteristics

Type of interviewee	Number of interviewees	Organisations with whom interviewees were linked
**External stakeholders**	7<br>(4 female, 3 male)	NHS England<br>NHS Clinical Commissioning<br>General practice<br>National Association of Primary Care<br>New NHS Alliance<br>UK universities<br>Pharmacy-related media
**Internal stakeholders – organisational representatives**	8<br>(5 female, 3 male)	Royal Pharmaceutical Society<br>Pharmaceutical Services Negotiating Committee<br>National Pharmacy Association<br>Company Chemists' Association<br>Pharmacists' Defence Association[1]<br>Association of Pharmacy Technicians UK<br>Pharmaceutical industry
**Internal stakeholders – community pharmacists**[2,3]	10<br>(2 female, 8 male)	Independent community pharmacy (n=7)<br>Multiple community pharmacy (n=7)<br>Locum pharmacist (n=4)<br>Local pharmaceutical committees (n=2)<br>Pharmacy education bodies (n=2)

[1]Two interviewees linked to the Pharmacists' Defence Association were interviewed

[2]A multiple community pharmacy contractor was defined as consisting of six pharmacies or more; contractors with five or fewer pharmacies were regarded as independent.

[3]Some community pharmacists were linked to more than one type of organisation.

Table S4: Characteristics of interviewees from all backgrounds

ID number	Organisational stakeholders			Community pharmacist internal stakeholders				
	External	Internal	Gender	Independent community pharmacy[1]	Multiple community pharmacy [1]	Locum	Gender	Region in England
1				✓	✓	✓	Male	North
2		✓	Male					
3		✓	Female					
4		✓	Male					
5		✓	Male					
6				✓	✓	✓	Female	Midlands and East
7				✓			Male	North
8	✓		Female					
9		✓	Female					
10				✓			Male	Midlands and East
11		✓	Female					
12	✓		Male					
13				✓	✓		Male	North
14				✓			Male	South
15	✓		Male					
16	✓		Female					
17	✓		Female					
18		✓	Female					
19					✓		Male	South
20	✓		Male					
21					✓	✓	Female	South
22				✓	✓	✓	Male	North, Midlands and East
23		✓	Female					
24					✓		Male	North
25	✓		Female					
**TOTAL**	**7**	**8**	**Male (n=6) Female (n=9)**	**7**	**7**	**4**	**Male (n=8) Female (n=2)**	**-**

[1]A multiple community pharmacy contractor was defined as consisting of 6 pharmacies or more; contractors with 5 or fewer pharmacies were regarded as independent (General Pharmaceutical Services in England 2008/09 - 2018/19, NHS Digital).
