# Supplementary material for: Professional stakeholders’ expectations for the future of community pharmacy practice in England: a qualitative study
Source: BMJ Open. 2023 Oct 16;13(10):e075069. doi: 10.1136/bmjopen-2023-075069 (PMC10582972; doi:10.1136/bmjopen-2023-075069)
Supplement: Author's manuscript [file bmjopen-2023-075069.draft_revisions.pdf]

BMJ Open is committed to open peer review. As part of this commitment we make the peer review history of every article we publish publicly available.

When an article is published we post the peer reviewers' comments and the authors' responses online. We also post the versions of the paper that were used during peer review. These are the versions that the peer review comments apply to.

The versions of the paper that follow are the versions that were submitted during the peer review process. They are not the versions of record or the final published versions. They should not be cited or distributed as the published version of this manuscript.

BMJ Open is an open access journal and the full, final, typeset and author-corrected version of record of the manuscript is available on our site with no access controls, subscription charges or pay-per-view fees (http://bmjopen.bmj.com).

If you have any questions on BMJ Open's open peer review process please email info.bmjopen@bmj.com

Professional stakeholders' expectations for the future of community pharmacy practice in England: a qualitative study

Journal:	*BMJ Open*
Manuscript ID	bmjopen-2023-075069
Article Type:	Original research
Date Submitted by the Author:	25-Apr-2023

Complete List of Authors:	Paloumpi, Evgenia; University of Bath, Department of Life Sciences<br>Ozieranski, Piotr; University of Bath, Department of Social & Policy Sciences<br>Watson, Margaret; University of Strathclyde<br>Jones, Matthew; University of Bath, Department of Life Sciences

Keywords:	Primary Care < Primary Health Care, Health policy < HEALTH SERVICES ADMINISTRATION & MANAGEMENT, Organisation of health services < HEALTH SERVICES ADMINISTRATION & MANAGEMENT, PUBLIC HEALTH, QUALITATIVE RESEARCH

SCHOLARONE™
Manuscripts

I, the Submitting Author has the right to grant and does grant on behalf of all authors of the Work (as defined in the below author licence), an exclusive licence and/or a non-exclusive licence for contributions from authors who are: i) UK Crown employees; ii) where BMJ has agreed a CC-BY licence shall apply, and/or iii) in accordance with the terms applicable for US Federal Government officers or employees acting as part of their official duties; on a worldwide, perpetual, irrevocable, royalty-free basis to BMJ Publishing Group Ltd ("BMJ") its licensees and where the relevant Journal is co-owned by BMJ to the co-owners of the Journal, to publish the Work in this journal and any other BMJ products and to exploit all rights, as set out in our licence.

The Submitting Author accepts and understands that any supply made under these terms is made by BMJ to the Submitting Author unless you are acting as an employee on behalf of your employer or a postgraduate student of an affiliated institution which is paying any applicable article publishing charge ("APC") for Open Access articles. Where the Submitting Author wishes to make the Work available on an Open Access basis (and intends to pay the relevant APC), the terms of reuse of such Open Access shall be governed by a Creative Commons licence – details of these licences and which Creative Commons licence will apply to this Work are set out in our licence referred to above.

Other than as permitted in any relevant BMJ Author's Self Archiving Policies, I confirm this Work has not been accepted for publication elsewhere, is not being considered for publication elsewhere and does not duplicate material already published. I confirm all authors consent to publication of this Work and authorise the granting of this licence.

For peer review only

Professional stakeholders’ expectations for the future of community pharmacy practice in England: a qualitative study

Evgenia Paloumpi[a], Piotr Ozieranski[b], Margaret C Watson[c] and Matthew D. Jones[d] (corresponding author)

[a]Department of Life Sciences, University of Bath, Bath, England. evina.paloumpi@gmail.com

[b]Department of Social & Policy Sciences, University of Bath, Bath, England. P.Ozieranski@bath.ac.uk

[c]Strathclyde Institute of Pharmacy and Biomedical Sciences, University of Strathclyde, Glasgow, Scotland. margaret.watson@strath.ac.uk

[d]Department of Life Sciences, University of Bath, Bath, England. M.D.Jones@bath.ac.uk, telephone: +44 1225 383829

For peer review only - http://bmjopen.bmj.com/site/about/guidelines.xhtml

Abstract

Objectives: To explore the views of professional stakeholders on the future of community pharmacy services in England. Specific objectives related to expectations of how community pharmacy services will be provided by 2030 and the factors that will influence this.

Design: Qualitative, using semi-structured interviews in person or via telephone or Skype. The topic guide was informed by a recent policy review and the Walt and Gilson policy framework. Transcripts were analysed using inductive thematic analysis.

Setting: England

Participants: Internal and external health-sector stakeholders, including policymakers, commissioners, representatives of healthcare professionals and community pharmacists. Interviewees were identified using stakeholder mapping

Results: In total, 25 interviews were completed (7 external stakeholders and 18 internal stakeholders, of which 10 were community pharmacists). Community pharmacy was recognised as having a key role in expanding capacity within the health system *("…pharmacy is the obvious person to take on those roles…")*, particularly for long-term condition management (e.g. adherence, reducing polypharmacy, monitoring), urgent care (e.g. minor illnesses) and public health (including mental health). For these contributions to be developed and optimised, greater integration and collaboration with general practices will be needed (*"…there is no room for isolationism in pharmacy anymore…"*), as well as use of technology in a patient-centred way and full access to health records. These developments will require workforce development together with appropriate commissioning and contractual arrangements. Community pharmacy is currently undervalued (*"…the complete*

For peer review only - http://bmjopen.bmj.com/site/about/guidelines.xhtml

misunderstanding by senior Government officials is very scary") and recent investment in general practice pharmacists rather than community pharmacy was seen as a missed opportunity.

Conclusions: Community pharmacy as a sector could and should be developed to increase the capacity of the health service to address its current challenges. Numerous modifications are required from a range of stakeholders to create the environment in which these changes can occur.

For peer review only

Strengths and limitations of this study

- The key strength of this study is its examination of the development of community pharmacy services in England in their entirety (rather than individual services) from the perspective of a wide variety of systematically selected interviewees, making it more representative and generalisable than some previous studies.
- The inability to recruit stakeholders from the Government and organisations that represent the public limits the breadth of perspectives included.
- The addition of pharmacists working in other sectors (e.g. GP pharmacists) could have added useful insights.

Funding statement

This work was supported by Sigma Pharmaceuticals via the Bharat Shah PhD Scholarship. The funder had no role in the study design, the collection, analysis and interpretation of the data, the writing of the report and the decision to submit the article for publication.

For peer review only - http://bmjopen.bmj.com/site/about/guidelines.xhtml

Competing interests

None declared.

Data sharing statement

Not applicable due to lack of participant consent.

Word count

5837 words

Keywords

Community pharmacy services; England; stakeholder participation.

For peer review only

For peer review only - http://bmjopen.bmj.com/site/about/guidelines.xhtml

1.0 Introduction

The National Health Service (NHS) in England (the largest and most populous part of the United Kingdom) is currently facing unprecedented work pressures. In primary care, increased patient demand combined with a reduction in the number of general practitioners (GPs), poor morale and decreased real-terms funding per capita has led many to talk of a system in "crisis"[1]. The secondary care sector is facing similar challenges[2].

Community pharmacies are one of four fundamental sectors of primary care in England[3.] They are easily accessible premises where qualified healthcare professionals supply medicines and provide medicines-related and public health services. There is evidence of the effectiveness of community pharmacy services to manage long-term conditions[4], minor ailments[5] and promote public health[6]. Greater use of community pharmacy has therefore been identified as an important way to address current NHS challenges[7]. However, despite the substantial contribution that community pharmacies make to the NHS throughout England, particularly during the Covid-19 pandemic, the sector is experiencing sustained underfunding[8], increased rates of closure[9] and growing competition from online pharmacies[10].

In addition, since 2015 there has been a large increase in the number of 'general practice pharmacists' in England[11]. This is a distinct role to that of community pharmacists, responsible for various tasks in general practices, such as structured medication reviews and medicines reconciliation. The impact of GP pharmacists on community pharmacy has not been widely explored.

For peer review only - http://bmjopen.bmj.com/site/about/guidelines.xhtml

A tipping point is being reached for community pharmacy in England, with an urgent need for a national vision and strategy[12]. A multi-stakeholder perspective combined with a system-wide approach is needed to ensure that strategic developments reflect not only need but capabilities. There has been no recent exploration of stakeholders' visions for the future of the community pharmacy and earlier consultations have focused on singular activities.

This study explored the views of health-sector stakeholders on the short-to-midterm (10-year) future of community pharmacy in England. Specific objectives were to explore:

- Expectations of how community pharmacy services will be provided by 2030;
- Factors that will influence future service development and delivery;
- Opinions of how community pharmacy services should ideally be provided;
- Facilitators and barriers to the development these ideal community pharmacy services.

2.0 Methods

A qualitative interview design was chosen to allow in-depth exploration of stakeholders' views. The Research Ethics Approval Committee for Health (REACH) of University of Bath (reference EP 17/18 204) approved the study in August 2018. Reporting is in accordance with the Consolidated Criteria for Reporting Qualitative Research (Table S1, supplementary material).

For peer review only - http://bmjopen.bmj.com/site/about/guidelines.xhtml

2.1. Interviewees and recruitment

Both internal and external stakeholders were recruited. External stakeholders were representatives of non-pharmacy organisations. Internal stakeholders were either current community pharmacists or representatives of pharmacy organisations. Internal and external organisational representatives were recruited by direct invitation, whereas community pharmacists were recruited via advertisement. Interviewees gave written or audio informed consent.

Stakeholder mapping was used to systematically identify and invite a broad range organisational representatives (such as policymakers, commissioners and representatives of patients or healthcare professionals) to participate[13]. An initial list of stakeholders was constructed using a policy review[14] and research team expertise. A snowballing approach was also adopted to supplement any underrepresented stakeholder categories. Stakeholder profiles (career history, position, interest and influence) were created using publicly accessible information. These were used to select stakeholders to invite to interview. Up to five stakeholders were identified and ranked within each organisation. The highest ranked stakeholder was sent a written invitation, with telephone follow-up if needed. If a stakeholder declined, the next ranked individual from their organisation was invited. This process continued until a representative of every organisation been interviewed or all the identified stakeholders from an organisation had declined their invitation.

For peer review only - http://bmjopen.bmj.com/site/about/guidelines.xhtml

Community pharmacists were eligible for inclusion if they worked regularly in a community pharmacy in England and were identified using purposive sampling, to obtain a diverse sample based on a sampling matrix including type of community pharmacy, locum status, gender and geographical region. The study was advertised to community pharmacists via the Royal Pharmaceutical Society's (i.e. the professional body for pharmacy) online forums, local pharmaceutical committees, the research team's networks and social media. Every eligible person who responded to these advertisements was interviewed.

2.2. Data collection

Interviews were carried out by one trained researcher (EP) face-to-face, by telephone or Skype, to provide flexibility and increase recruitment[15]. A topic guide (supplementary material) was developed based on a recent policy review that employed the Walt and Gilson policy framework[14] and discussion within the research team. One pilot interview was conducted. Interviews were audio-recorded, transcribed verbatim and checked for accuracy and anonymity. The interviewer made notes of key points and observations from the interview.

2.3. Data analysis

Interview transcripts were analysed using inductive reflexive thematic analysis, which is suitable for applied health research aiming to inform the development of policy[16]. Analysis was iterative, based on a six-phase process: data familiarisation; generating initial codes; developing themes; reviewing themes; defining and naming themes; writing the analysis[16]. Initial manual coding was then interpreted to generate initial themes and subthemes using

For peer review only - http://bmjopen.bmj.com/site/about/guidelines.xhtml

NVivo v.12 software. Themes were iteratively refined for cohesiveness using a thematic map, before the final themes were named and defined. These processes were led by one researcher (EP), with regular discussion and agreement with the team.

2.4 Patient and public involvement statement

Patients and the public were not involved in this study. A separate study examining the perspectives of members of the public has been completed and will be reported.

3.0 Results

3.1 Interviewee characteristics

In total, 46 stakeholders were invited and 25 interviews were completed between November 2018 and April 2019. There were seven external stakeholders from seven organisations and eighteen internal stakeholders. Of these, eight were organisational representatives (from seven organisations) and ten were community pharmacists (Tables S2 and S3, supplementary material). Stakeholders representing three patient organisations, two state policy making organisations, two pharmacy representative organisations and three organisations representing other healthcare professions were invited, but did not participate. Interview duration varied from 35 to 75 minutes.

3.2 Overview of themes

Four themes with 11 sub-themes were generated from the analysis (Table 1). Each of these will be discussed in order of dominance.

For peer review only

For peer review only - http://bmjopen.bmj.com/site/about/guidelines.xhtml

Table 1: Themes and sub-themes generated from the analysis.

Theme	Sub-theme
Creating collaborations for wider pharmacy services provision from community pharmacy	Collaboration: expected, ideal and a facilitator
	Existing relationships with general practices
Integrating new technology into pharmacy services	Technology expected to change community pharmacy practice
	Making technology work for patients
	Information-sharing technology
Building workforce competency	Increasing responsibilities with a decreasing workforce
	Enhancing the skills of the workforce
	Role of pharmacy technicians
Future community pharmacy services	Community pharmacists' role in managing chronic conditions, urgent care and prevention
	Opportunities for further community pharmacy development
	Creating the environment for future developments

3.3 Theme 1: Collaboration for wider pharmacy services provision from community pharmacy

Subtheme: Collaboration: expected, ideal and a facilitator

> *"There is a coming together of health and social care…and I think pharmacy has to be in that tent…I don't see any future…for a pharmacy as a kind of standalone… there is no room for isolationism in pharmacy anymore it has to be seen as part of a wider team."*

Interviewee ID12 – external stakeholder (GP)

For peer review only

For peer review only - http://bmjopen.bmj.com/site/about/guidelines.xhtml

Interviewees expected future community pharmacy services to be provided collaboratively with other healthcare professionals and voluntary organisations. They envisaged more integration between professions with greater awareness of other professionals' skills and an understanding that services could be provided differently. Interviewees expected community pharmacists to work within local groups of general practices (known as primary care networks in England) and viewed current involvement as an encouraging step towards integration into primary care. Interviewees also described community pharmacists' strategic involvement in local and national structures as part of an ideal model of care. They emphasized that a community pharmacy culture change that promotes collaborative working would be crucial for patient care and the sector's survival.

Subtheme: Existing relationships with general practices

> *"…patients speak the language of medicines in terms of 'the blue ones' or 'the pink ones'… community pharmacists…straddle a divide between how patients think and talk and how the system thinks and talks…I deal with medicines every day of the week but somebody who is sat at their desk [GP pharmacist] wouldn't have a clue where they start with that."*

Interviewee ID14 – community pharmacist

For peer review only - http://bmjopen.bmj.com/site/about/guidelines.xhtml

There were contrasting opinions about the role of GP pharmacists in supporting community pharmacy services: external stakeholders viewed GP pharmacists as facilitators, but community pharmacists referred to them as a barrier. Internal stakeholders were disappointed that NHS England had invested mostly in GP pharmacists rather than the existing community pharmacy workforce, describing this as a missed opportunity. There was concern over a division between 'clinical' GP pharmacists and 'community' pharmacists, and some thought that community pharmacists were better placed to support patients.

> *"You only need to look at the debacle with the flu service…both GPs and pharmacists think they are in competition with each other and rather than working collaboratively…for the whole population..."*

Interviewee ID10 – community pharmacist

Community pharmacists described competitive *"them and us"* (Interviewee ID20) relationships with GPs, resulting from conflicting financial incentives (e.g. competition between community pharmacies and GP practices to vaccinate the same pool of patients against influenza) and poor understanding of pharmacists' skills (e.g. a reluctance by some GPs to share health records due to the view that pharmacists are not trained to protect confidential information). However, some believed that GPs valued community pharmacists and were optimistic about future relationships.

For peer review only - http://bmjopen.bmj.com/site/about/guidelines.xhtml
For peer review only

Community pharmacists perceived GPs as dominating primary care, mentioning *"lack of parity"* (Interviewee ID14) in the distribution of services and highlighting the need to align the medical and pharmaceutical NHS contracts. Overall, the GP contract and workload pressures in general practice were the most influential factors driving pharmacy service development, as the capabilities of community pharmacy were only discussed by a minority of interviewees.

3.4 Theme 2: Integrating technology into pharmacy services

Subtheme: Technology expected to change community pharmacy practice

> *"If we can use IT robotics just to take care of certain aspects which are more tedious and laborious…the pharmacists will have much more time to devote to patient care."*
>
> Interviewee ID15 – external stakeholder

New technology was seen as integral to future pharmacy services and healthcare in general. This was attributed to patient expectations, the entry of technological companies into the medicines market and the Government's intentions.

Interviewees described a 'hub and spoke' model, with automated dispensing of medicines in a central location, away from community pharmacy premises. This was expected and perceived as ideal, and was usually viewed as enabling a change from a medicines supply

For peer review only - http://bmjopen.bmj.com/site/about/guidelines.xhtml

model to one based on an enhanced clinical role. However, community pharmacists expressed concerns about delays to patient care and increased costs.

Home deliveries of medicines and increasing use of online pharmacies were commonly connected and were expected to increase, driven by convenience. Use of artificial intelligence to support patient decision-making, accurate prescribing and to reduce dispensing errors was also discussed, mainly by external stakeholders.

> *"… by 2030 Amazon could be delivering peoples' prescriptions door-to-door so where does pharmacy fit, if pharmacy hasn't got a more defined and a more developed clinical care role then, where is pharmacy?"*
>
> Interviewee ID12 – external stakeholder

Many highlighted the impact that these developments might have on the role of community pharmacists. Due to the risk of large wholesale companies dominating the medicines supply market, it will be necessary to establish a role based on supporting patients. A systematised, transactional and supply-only model for community pharmacy lacking knowledge of regular customers would have adverse consequences for patients.

For peer review only - http://bmjopen.bmj.com/site/about/guidelines.xhtml

Subtheme: Making technology work for patients

> *"…wearable technology…clinicians to deal remotely with the way that you are feeling…the fact that you are unwell can be detected at distance and then dealt with either at distance or…face-to-face..."*

Interviewee ID4 – internal stakeholder

> *"But it's how patients use that medicine and how they become effective with the medicine that is going to require the clinical expertise of the community pharmacist and that clinical expertise has to be done really face-to-face so that you can see the patient more effectively."*

Interviewee ID10 – community pharmacist

All interviewees described the growing role that healthcare applications (apps) and devices would have in pharmacy services, expecting transformed patient interaction for the supply of medicines, diagnosis and review of patient data. Future patients will require community pharmacists to understand and assist with these sources of information. Interviewees expected increased use of online consultations driven by patient expectations and preference but highlighted the importance of maintaining face-to-face contact with patients. Community pharmacists expressed concerns that face-to-face consultations were more effective. A 'mixed model' of digital and face-to-face communication was seen as ideal.

For peer review only

For peer review only - http://bmjopen.bmj.com/site/about/guidelines.xhtml

"…we have to be careful we don't generate technology poverty, so we don't want to exclude a sector of the population."

Interviewee ID23 – internal stakeholder

Interviewees were cautious about how technology might exclude older generations. Community pharmacists were considered fundamental for preventing such inequalities and promoting familiarity with technology.

Subtheme: Information-sharing technology

"… I think once that link [read and write access to health records] is…established…in terms of what we can then do…it opens it up…anything and everything really."

Interviewee ID21 – community pharmacist

All interviewees referred to community pharmacist access to health records as a crucial facilitator for pharmacy services and for some community pharmacists this was ideal. It would allow seamless care, enable community pharmacists to manage long-term conditions more effectively and relieve GP workload. Current read-only access to Summary Care Records (an electronic summary of patients' key clinical information) was beneficial, but the ability to add information is required. Some interviewees thought this would enable

For peer review only - http://bmjopen.bmj.com/site/about/guidelines.xhtml

community pharmacists to communicate medication changes, thus ensuring their advice is followed and providing GPs with an accurate picture of their interventions.

> *"The personalised care and the genomics is something that…pharmacists…need to make themselves really familiar with because that is the future..."*

Interviewee ID18 – internal stakeholder

Interviewees expected new sources of patient data from developments such as pharmacogenomics. Professionals will need to understand and interpret these data, for which community pharmacists should be prepared.

3.5 Theme 3: Building workforce competence

Subtheme: Increasing responsibilities with a decreasing workforce

> *"The current Government agenda in relation to the closure of high street pharmacy, …I think that was scary…I have had a very senior person say to me we need to close these pharmacies because all they do is sell shampoo. I think the complete misunderstanding by senior Government officials is very scary."*

Interviewee ID8 – external stakeholder

Most community pharmacists expressed insecurity about their future and felt their value was not recognised due to funding cuts and policies aiming to reduce the number of

For peer review only - http://bmjopen.bmj.com/site/about/guidelines.xhtml

pharmacies. Lack of career development and incentives for community pharmacists was another factor contributing to a declining workforce. Community pharmacists referred to a tired, disillusioned and demotivated workforce working in tough conditions, warning that many might choose alternative careers (e.g. GP pharmacy), thus further reducing the workforce.

Nevertheless, interviewees also expected that community pharmacy responsibilities will increase, referring to community pharmacies becoming an increasingly clinical environment with a reduced medicines supply function. Some community pharmacists expected a transfer of services from GP practices. Therefore, interviewees envisaged that in 2030 there would be more than one pharmacist per pharmacy. For some, this was ideal, providing time for patient interaction and clinical roles.

For peer review only

For peer review only - http://bmjopen.bmj.com/site/about/guidelines.xhtml

Subtheme: Enhancing the skills of the workforce

> *"It is a no brainer to me that all pharmacists should be independent prescribers as an absolute pre-requisite…I think the independent prescribing as it currently stands is patchy it's not many community pharmacists doing it and it is not joined up, it is not really getting us anywhere."*
>
> Interviewee ID12 – external stakeholder

> *"I think at the moment the training for pharmacists working in community pharmacy is very piecemeal and there is…the problem of…getting released from work..."*
>
> Interviewee ID3 – internal stakeholder

It was clear that more community pharmacists should become independent prescribers with appropriate funding and training support. Prescribing was a facilitator that would allow community pharmacists to improve long-term condition management, run clinics for specific diseases and release more GP time for complex cases. External stakeholders supported the competence of pharmacists for this role.

Interviewees referred to the need to train the community pharmacy workforce for such responsibilities, including physical examinations, clinical assessment, consultation skills and data management. Many internal stakeholders believed there were skill differences in these areas between generations of pharmacists. Training alongside other healthcare

For peer review only

For peer review only - http://bmjopen.bmj.com/site/about/guidelines.xhtml

professionals, particularly doctors, will improve mutual understanding. Most community pharmacists described significant barriers to training, including time, funding and accessibility. They believed government expectations were unrealistic in the absence of protected time or funding, highlighting the need for more resources.

"Some pharmacists are their own worst enemy…a lot of them want to hold onto the dispensing process…they're quite happy with how things are..."

Interviewee ID1 – community pharmacist

Interviewees also referred to the sector's resistance to change as one of the biggest barriers to pharmacy services, especially those who might prefer the current situation or be reluctant to risk change to a profitable business. Some stakeholders believed that the businesses that were unwilling to change would suffer. Consequently, some external stakeholders highlighted the importance of understanding community pharmacists' values and motivations. Some internal stakeholders perceived younger pharmacists as advocating for a clinical role, with the contrasting dispensing-focus of some older pharmacist's seen as inconsiderate of their legacy to the future generations.

For peer review only

For peer review only - http://bmjopen.bmj.com/site/about/guidelines.xhtml

Subtheme: Role of pharmacy technicians

> *"…there are massively differing standards of technicians. There are some that I would trust to run a pharmacy with my life and there are others that I would not let near a pharmacy with a bargepole…"*
>
> Interviewee ID21 – community pharmacist

Interviewees recognised pharmacy technicians as underused and suggested making greater use of their skills. A variety of roles were suggested, such as enhancing their role in medication supply to increase cost-effectiveness and release more clinical time for pharmacists. Additional roles included simple consultations, smoking cessation, weight management, influenza vaccination and domiciliary visits. Training will be essential, considering the existing variability in the skills of pharmacy technicians. However, funding for such training was a concern for some community pharmacists, as recent funding cuts had impacted on investment in technician development.

For peer review only

For peer review only - http://bmjopen.bmj.com/site/about/guidelines.xhtml

3.6 Theme 4: Future community pharmacy services

Subtheme: Community pharmacists' role in managing long-term conditions, urgent care and prevention

> *"These [long-term conditions]…cannot be dealt with by general practice alone and therefore pharmacy is the obvious person to come on and take on those roles so I can see that aspect growing significantly."*
>
> Interviewee ID20 – external stakeholder

Interviewees expected that community pharmacists' role in the management of long-term conditions would be enhanced in the future, to reduce medicine-related hospital admissions and release GP time. Community pharmacist support of medication adherence was already beneficial and there are opportunities for improving medicines safety, polypharmacy, monitoring and screening services. Drivers for these developments included current success, an existing team of capable staff, and rising multimorbidity, life expectancy and polypharmacy. Some community pharmacists viewed a more prominent role in long-term condition management as ideal, envisaging a future where they would be adequately resourced to monitor and manage medicines for long-term conditions such as asthma, diabetes and cardiovascular disease. However, some interviewees did not foresee further developments for community pharmacy in long-term condition management, with one warning of a *"difficult journey"* (interviewee ID12 – external stakeholder) requiring collaborations with other healthcare professionals.

For peer review only - http://bmjopen.bmj.com/site/about/guidelines.xhtml

"There would need to be a change in the…training that a pharmacist gets where it becomes more of a hybrid between being a pharmacist and being a doctor-physician's assistant…without that additional clinic support it's difficult to see how emergency services offered through community pharmacies would grow much beyond the current minor ailment-type model."

Interviewee ID20 – external stakeholder

Further developments in urgent and emergency care were also expected. Interviewees identified this as an area where the community pharmacist's role has progressed and will continue to develop quickly. Examples included in the NHS 111 triage system, the NHS Urgent Medicine Supply Advanced Service and encouraging local services. Community pharmacy will be involved in reducing visits to emergency departments and thus reducing costs. This was seen as one of the most likely developments, due to its strong presence in national policies. However, interviewees did not view community pharmacies as emergency drop-in centres, as they can only respond to a specific set of conditions.

Community pharmacies were described as the first port of call for minor illnesses, such as skin complaints and eye infections. However, some internal stakeholders believed that community pharmacies could do more with a nationally commissioned minor ailments service. Independent prescribing was also a facilitator for greater involvement in this area. Providing care for minor ailments was perceived as limiting community pharmacies' ability to provide more advanced services. In addition, interviewees of all types questioned the competence of some pharmacists to provide advanced urgent care.

For peer review only - http://bmjopen.bmj.com/site/about/guidelines.xhtml

For peer review only

"I would expect to see definite growth…as much as NHS England talks about the prevention agenda they'll start to engage with community pharmacy which is the largest single healthcare provider of both patients and people before they get ill"

Interviewee ID14 – community pharmacist

The community pharmacy network was also expected to have a prominent future role in promoting wellness and disease prevention, because of existing public health services, accessibility, well-equipped pharmacy teams and the advantage of providing care to healthy individuals. However, it was thought that community pharmacy would require improved remuneration for this role.

Subtheme: Opportunities for further community pharmacy development

"…pharmacies become health hubs in the future and if they did…they need to be bigger than they are today…they need to have a different look, feel, access information."

Interviewee ID9 - internal stakeholder

There was a belief that community pharmacies were well located to serve the needs of their communities and should ideally become centres for advice and health support in one location. However, some internal stakeholders suggested that pharmacies need more space, with less emphasis on retail and a more professional environment.

For peer review only - http://bmjopen.bmj.com/site/about/guidelines.xhtml

> *"My biggest one [hope] would be that community pharmacy would be recognised as a part of a social solution and not just a clinical solution and would they be able to sell itself…within an integrated care system…thinking about what…social value could be exploited out of community pharmacy in terms of people, place and planet."*
>
> Interviewee ID8 – external stakeholder

Interviewees envisaged a future where community pharmacy's role included tackling health inequalities, including ensuring that care was accessible in socially deprived areas. This might involve a system of 'prescriptions' for pharmacy services and involvement in social prescribing schemes.

> *"Absolutely there is opportunity for us to help people with mental health needs both from a chronic and low level to the more acute; there's definitely stuff that community pharmacy can do."*
>
> Interviewee ID5 – internal stakeholder

Mental health care in community pharmacies was presented as an opportunity, due to increasing demand and national recognition. Some community pharmacists described mental health services that were currently provided included advice, reassurance and signposting. Community pharmacies were seen as advantageous for providing mental health care due to their convenient location, local knowledge and patient relationships that could facilitate early detection of symptoms and initial assessment. Interviewees proposed a

For peer review only - http://bmjopen.bmj.com/site/about/guidelines.xhtml

range of interventions that might be provided, including signposting, lifestyle advice, medication adherence monitoring, adverse effect management and provision of specialist services such as Cognitive Behavioural Therapy in consultation rooms. Some strongly believed that antidepressants should be included in the NHS New Medicines Service, describing their exclusion (at the time of data collection) as *"appalling"* (ID23). However, some interviewees described mental health services as the least likely development, due to lack of resources and training needs.

Community pharmacists described the success of influenza vaccinations in community pharmacies, which are convenient and reduce GP workload. Based on this, interviewees described opportunities to expand community pharmacists' role in vaccination and administering injections including denosumab, vitamin B12, antipsychotic depot injections and corticosteroids, although others were reluctant to see this.

Subtheme: Creating the environment for future developments

> *"We are not really too concerned about the level of care that we give to our patient and again that purely goes down to the way we are funded…if we were to pay pharmacies more for hitting quality targets, for looking after patients…I think then we would see better care..."*

Interviewee ID22 – community pharmacist

For peer review only

For peer review only - http://bmjopen.bmj.com/site/about/guidelines.xhtml

Interviewees could not envisage an ideal pharmacy services model without greater funding. They argued that reimbursement based on dispensing volume instead of service quality was not fit for purpose and hindered the community pharmacy workforce in using its clinical expertise to fully benefit patients. Some community pharmacists also expressed disappointment at providing clinical services without being reimbursed.

> *"I think one of the challenges for pharmacy is consistency and continuity…if we've got lots of disparate offerings from lots of different pharmacies that confuses patients, whereas what I want to see is a more standardised nationalised commissioning of services."*
>
> Interviewee ID23 – internal stakeholder

The need for support from commissioners at all levels was acknowledged. Some community pharmacists described feeling lost due to the lack of a coherent approach to commissioning, referring to NHS England's 'Community Pharmacy Clinical Services Review' as abandoned and to unfulfilled plans for newly commissioned services. They believed that long-term plans would allow investment, highlighting the influence of local commissioners and the importance of consistency.

External stakeholders from a commissioning background stated that a coherent commissioning model was one of the biggest challenges for pharmacy services, highlighting the current model's complexity. Commissioning for community pharmacy was described as

For peer review only - http://bmjopen.bmj.com/site/about/guidelines.xhtml

fragmented compared with commissioning of other primary care providers and more integrated thinking was required.

> *"Improve our image with the community…because if you ask a patient 'well what do pharmacists do?' they just think we pop up in the back of a pharmacy, wear lab coats and hand them out…this is one of the reasons why other healthcare professionals don't even trust us as much as they should. It is because our professional body hasn't done enough to demystify our skills."*
>
> Interviewee ID22 – community pharmacist

Many community pharmacists felt dissatisfied with their representation. They referred to the lack of a *"true voice for pharmacists"* (ID14) in influential bodies, at every level of the NHS, and to other healthcare professionals and the public.

> *"… in my 13 years as a qualified pharmacist, I have not seen anything from either NHS England, the Chief Pharmaceutical Officer, the Department of Health and Social Care or Her Majesty's Government, I have seen no action that actually backs up their nice words for community pharmacy. I don't see that they trust community pharmacy to do anything other than simply dole out medicines. In fact, that is the precise term that NHS England Chief Executive said, why are we spending all this money doling out*

For peer review only - http://bmjopen.bmj.com/site/about/guidelines.xhtml

medicines which was really quite an offensive thing to say but that is all the NHS sees us for."

Interviewee ID24 – community pharmacist

It was important to have pharmacists working in strategic positions in clinical commissioning groups (since replaced by integrated care boards) and the Department of Health and Social Care to drive future changes. Support from other representatives such as the Royal Pharmaceutical Society, Pharmaceutical Services Negotiating Committee and Chief Pharmaceutical Officer was considered essential. Interviewees referred to inadequate national support and leadership for community pharmacy. Many internal stakeholders criticised the Royal Pharmaceutical Society as a representative body, stating that it did not advocate beyond medicines supply and questioned whether it would be fit for purpose in the future. Some external stakeholders highlighted the need for the pharmacy profession to speak with one voice. All of this led to poor recognition of the sector by the state. Interviewees with policymaking experience were still unaware of the role of community pharmacy and highlighted the need to define the role of community pharmacy in policy. There was a disconnect between the governmental and community pharmacy view of the sector's strengths, with the need for the government to trust community pharmacy and see it as "integral".

Evidence was considered an important factor that would contribute to better recognition for community pharmacy. Interviewees from all backgrounds acknowledged the importance of strengthening the evidence base for community pharmacist interventions through

For peer review only

For peer review only - http://bmjopen.bmj.com/site/about/guidelines.xhtml

enhanced research and intervention recording. Creating evidence responsive to NHS challenges was significant. An external stakeholder with commissioning experience referred to the need for *"iterative"*, *"applicable at a wider scale," "affordable" and "timely"* evidence (ID17), while another identified a *"huge research gap"* (ID8) for community pharmacy interventions with an impact on social care.

Some suggested that all community pharmacy contractors work together to do this, with multiple chain pharmacies described as *"big data power houses"* (ID15). There were contrasting views regarding the influence of evidence. Most believed that pilot services, trials, local schemes and good practice could lead to change by replication at a national level. However, some community pharmacists did not share this belief.

3.7 Convergence and divergence between stakeholder types

There were large areas of convergence in the views discussed by external stakeholders and internal stakeholders (both organisational representatives and community pharmacists) (Figure 1). In particular, they related to community pharmacy services, technology and the workforce. There was also considerable divergence with only community pharmacists discussing how the sector felt insecure and undervalued and expressing concerns about technology and collaborative relationships.

For peer review only - http://bmjopen.bmj.com/site/about/guidelines.xhtml

[Insert Figure 1]

Figure 1: Areas of convergence and divergence in the views discussed by external stakeholders and internal stakeholders (both organisational representatives and community pharmacists).

4.0 Discussion

This study describes the views of a wide variety of systematically selected stakeholders' on how community pharmacy services may be provided in the short-to-midterm, in order to meet current NHS challenges. All types of stakeholder anticipated increased provision of services for long-term conditions, urgent care and public health, and viewed collaboration with general practice, greater use of technology, workforce development, and financial and contractual reforms as facilitators to enable this. Several barriers to these developments were also identified, which were especially apparent in the varying perspectives of internal and external stakeholders. These included an insecure and demotivated workforce, poor relationships between community pharmacy and general practice, and inadequate and disunited leadership and representation.

Expectations that community pharmacists will provide services for long-term conditions, urgent care and public health are supported by evidence of the effectiveness of such interventions[4-6]. Since the interviews were completed, a number of new nationally commissioned services in these areas have been introduced, including the Discharge Medicines Service, the Community Pharmacist Consultation Service (with GP referrals), the Smoking Cessation Service and the Hypertension Case Finding Service[17]. These services demonstrate the importance of greater collaboration between community pharmacy and

For peer review only - http://bmjopen.bmj.com/site/about/guidelines.xhtml

general practice, as some require referral of patients between these two settings, and many were initially part of the Pharmacy Integration Fund (PhIF). This was established in 2016 to accelerate the integration of pharmacy services into the wider NHS[18]. The PhIF demonstrates the importance of nationally-led piloting and commissioning of pharmacy services, as this overcomes local difficulties in community pharmacy-GP relationships. More recently, the PhIF has funded a community pharmacy clinical lead in all areas of England to further integrate new pharmacy services into the wider NHS.

However, given the importance of collaboration, actions to overcome the difficult community pharmacy-GP relations described by interviewees will be important, especially as this has been reported before[19]. Moreover, this study found for the first time that such competition extends to relationships between community and GP pharmacists, although other intra-professional tensions, lack of awareness and concern about the financial viability of community pharmacy have been observed previously[20, 21]. The growing number of GP pharmacists means that GPs now have greater experience of the benefits of working with the pharmacy profession. This provides an opportunity for individual pharmacists and pharmacy organisations to improve local relationships with GPs, to the benefit of patients and the NHS. For example, interprofessional education is now a requirement in initial training for both pharmacists and doctors and has been shown to improve attitudes towards interprofessional collaboration[22].

Aspects of workforce development were also key facilitators identified in this study, particularly independent prescribing qualification for pharmacists and extended roles for

For peer review only - http://bmjopen.bmj.com/site/about/guidelines.xhtml

pharmacy technicians. Although a number of recent initiatives have provided funding for independent prescribing training for community pharmacists, in 2021 there was only one community pharmacist independent prescriber for every ten community pharmacies, an unchanged proportion from 2017[23]. All newly qualified pharmacists will be independent prescribers from 2026, but further initiatives are required to train the existing workforce.

Better use of automation and technology will be required to use a more skilled workforce efficiently. The NHS currently lacks the national IT infrastructure required to give all community pharmacists access to patients' full health records, which was a key facilitator of collaboration with GPs identified in this study. However, a number of local pilot schemes are underway, with a target for national access by March 2025[24]. Interviewees also identified automated dispensing as releasing community pharmacists to provide new services. While there is evidence of the safety, efficiency and cost-effectiveness of automated community pharmacy dispensing[25], this has not yet been widely adopted by community pharmacies in England.

Financial and contractual reforms to balance income from retail and cognitive activities were identified as important facilitators of future pharmacy services[26]. This is demonstrated by the rapid uptake of newly commissioned national services, such as the Community Pharmacist Consultation Service and the Hypertension Case Finding Service (over 98,000 and 113,000 consultations in October 2022, respectively)[27]. The national commissioning of pharmacy services is thus a strong driver for change, particularly as it means patients have access to a consistent range of services from most community pharmacies, encouraging

For peer review only - http://bmjopen.bmj.com/site/about/guidelines.xhtml

greater use. The PhIF model of national commissioning of services that have undergone a successful centrally funded pilot[18] should therefore be continued, to ensure consistent integration of community pharmacy with the wider NHS in all areas of England.

Such developments will only happen with strong leadership, from both the NHS and the community pharmacy sector. Both internal and external stakeholders were clear that community pharmacy leadership and representation is currently poor, particular because it is provided by multiple organisations that do not present a unified position. Stronger, unified leadership of community pharmacy is therefore also required.

4.1 Strengths and limitations

The key strength of this study is its examination of the development of community pharmacy services in England in their entirety (rather than individual services) from the perspective of a wide variety of systematically selected interviewees, making it more representative and generalisable than some previous studies. However, the inability to recruit stakeholders from the Government and organisations that represent the public limits the breadth of perspectives included. A separate study examining the perspectives of members of the public has been completed and will be reported. The addition of pharmacists working in other sectors (e.g. GP pharmacists) could have added useful insights. Despite the interviews being conducted in 2018-19, the results remain salient, if not more so, due to the intervening Covid-19 pandemic which has accelerated development of many of the topics discussed.

For peer review only - http://bmjopen.bmj.com/site/about/guidelines.xhtml

4.2 Recommendations

Based on these findings, the Department of Health and Social Care and NHS should implement policies that ensure that community pharmacy is fully integrated with the wider primary care system and can work collaboratively with other healthcare professionals (e.g. by enabling full access to health records). Clinical and contractual incentives for the primary care sector should be aligned to avoid creating competitive relationships between professions. In addition, the state should facilitate the use of automated dispensing in community pharmacy, to release pharmacist time for new services. Such services are likely to be related to long-term conditions, urgent care, minor ailments and public health, but may also include reducing health inequalities, providing vaccination and support for people with mental health problems. Commissioning of community pharmacy services should be reformed to create a less fragmented national system with adequate renumeration based on patient-orientated services rather than dispensing volume. Finally, policies are required to develop the skills of the community pharmacy workforce for both current and anticipated roles, with a particular focus on independent prescribing and pharmacy technicians.

Pharmacy representative organisations should ensure that the community pharmacy sector is represented effectively to multiple audiences (the Government, NHS, other professions and the public) and presents a unified voice and shared vision. They should especially advocate for the implementation of the policies recommended above and increase awareness of the role of community pharmacists amongst other healthcare professionals and the public. They should also promote a culture of participation in research to generate and publish an evidence-base for community pharmacy services that is responsive to NHS

For peer review only

For peer review only - http://bmjopen.bmj.com/site/about/guidelines.xhtml

needs. Finally, community pharmacy businesses and community pharmacists should be proactive in supporting these developments and need to take urgent action to support their insecure and demotivated workforce.

5.0 Conclusion

Stakeholders expect that in the future community pharmacists will help to address current NHS challenges by managing long-term conditions, providing urgent care and public health services. Collaboration with primary care and use of new technologies will be fundamental for facilitating these changes and ensuring they benefit patients. Numerous changes are required from the Government, NHS, pharmacy representative bodies, community pharmacy businesses and community pharmacists to create the environment in which these changes can occur.

Author contributions

EP: Conceptualization; Methodology; Project administration; Data curation; Formal analysis; Writing – original draft; Writing – original draft.

PO: Conceptualization; Methodology; Supervision; Writing - review & editing.

MW: Conceptualization; Methodology; Supervision; Funding acquisition; Writing - review & editing.

MDJ: Conceptualization; Methodology; Supervision; Funding acquisition; Writing – review & editing.

For peer review only - http://bmjopen.bmj.com/site/about/guidelines.xhtml

Acknowledgements

The authors would like to sincerely thank all the stakeholders who participated in the study and everyone who helped with their recruitment. The authors would also like to thank Dr Bharat Shah CBE and Sigma Pharmaceuticals. Dr E Paloumpi's PhD was funded by The Bharat Shah PhD Scholarship.

References

1. Razai MS and Majeed A. General practice in England: the current crisis, opportunities, and challenges. *Journal Ambulatory Care Management* 2022; 45: 135-139. DOI: 10.1097/JAC.0000000000000410.
2. Cooksley T, Clarke S, Dean J, et al. NHS crisis: rebuilding the NHS needs urgent action. *BMJ* 2023; 380: 1. 20230103. DOI: 10.1136/bmj.p1.
3. Baird B and Beech J. Community pharmacy explained https://www.kingsfund.org.uk/publications/community-pharmacy-explained (2020, accessed 20 January 2021 2021).
4. Evans CD, Watson E, Eurich DT, et al. Diabetes and cardiovascular disease interventions by community pharmacists: A systematic review. *The Annals of Pharmacotherapy* 2011; 45: 615-628.
5. Watson M, Holland R, Ferguson J, et al. *Community Pharmacy Management of Minor Illness (MINA study)*. 2014. London, UK.
6. Thomson K, Hillier-Brown F, Walton N, et al. The effects of community pharmacy-delivered public health interventions on population health and health inequalities: A review of reviews. *Prev Med* 2019; 124: 98-109. 2019/04/09. DOI: 10.1016/j.ypmed.2019.04.003.
7. Royal College of Emergency Medicine, Royal College of General Practitioners, Royal College of Physicians, et al. Rebuilding the NHS: better medical pathways for acute care 2022, https://www.rcplondon.ac.uk/projects/outputs/rebuilding-nhs-better-medical-pathways-acute-care-2022 (2022, accessed 31st January 2023).
8. Ernst and Young. *Impacts of current funding, policy and economic environment on independent pharmacy in England* 2020. London, UK.
9. Wickware C and Wilkinson E. The new abnormal: vulnerable patients hit by pharmacy closures. *The Pharmaceutical Journal*. 2020.
10. Wickware C. Online pharmacy dispensing volume grows by 45% in 2020, fuelled by COVID-19 pandemic. *Pharmaeutical Journal* 2021; 306. DOI: 10.1211/PJ.2021.1.78119.
11. Alshehri AA, Cheema E, Yahyouche A, et al. Evaluating the role and integration of general practice pharmacists in England: a cross-sectional study. *International Journal of Clinical Pharmacy* 2021; 43: 1609-1618. 20210602. DOI: 10.1007/s11096-021-01291-6.
12. Wright D, Twigg M, Family H, et al. *Independent Review of Community Pharmacy Contractor Representation and Support: "Providing best value for contractors"*. 2020. Norwich, UK.
13. Varvasovszky Z and Brugha R. How to do (or not to do) a stakeholder analysis. *Health Policy Plan* 2000; 15: 338–345.

For peer review only

For peer review only - http://bmjopen.bmj.com/site/about/guidelines.xhtml

14. Paloumpi E, Ozieranski P, Watson MC, et al. Professional and state policy on community pharmacy: a 10-year policy review and comparative analysis (2008-2017). *Exploratory Research in Clinical and Social Pharmacy* 2022: Submitted.
15. Harvey WS. Strategies for conducting elite interviews. *Qualitative Research* 2011; 11: 431-441. DOI: 10.1177/1468794111404329.
16. Braun V and Clarke V. Using thematic analysis in psychology. *Qualitative Research in Psychology* 2006; 3: 77-101. DOI: 10.1191/1478088706qp063oa.
17. Pharmaceutical Services Negotiating Committee. National pharmacy services, https://psnc.org.uk/national-pharmacy-services/ (2022, accessed 31st January 2023).
18. NHS England. Pharmacy Integration Programme, https://www.england.nhs.uk/primary-care/pharmacy/pharmacy-integration-fund/ (2023, accessed 1st February 2023).
19. Hindi AMK, Jacobs S and Schafheutle EI. Solidarity or dissonance? A systematic review of pharmacist and GP views on community pharmacy services in the UK. *Health and Social Care in the Community* 2019; 27: 565-598. 2018/07/27. DOI: 10.1111/hsc.12618.
20. Karampatakis GD, Patel N, Stretch G, et al. Community pharmacy teams' experiences of general practice-based pharmacists: an exploratory qualitative study. *BMC Health Services Research* 2020; 20: 431. 2020/05/20. DOI: 10.1186/s12913-020-05245-y.
21. Nabhani-Gebara S, Fletcher S, Shamim A, et al. General practice pharmacists in England: Integration, mediation and professional dynamics. *Research in Social and Administrative Pharmacy* 2020; 16: 17-24. 20190201. DOI: 10.1016/j.sapharm.2019.01.014.
22. Lapkin S, Levett-Jones T and Gilligan C. A systematic review of the effectiveness of interprofessional education in health professional programs. *Nurse Education Today* 2013; 33: 90-102. 20111222. DOI: 10.1016/j.nedt.2011.11.006.
23. Centre for Pharmacy Workforce Studies. *Community pharmacy workforce survey 2021*. 2022. Health Education England.
24. Wickware C. Patient records: when will community pharmacies get access? *Pharmaceutical Journal* 2022; 309. DOI: 10.1211/PJ.2022.1.153943.
25. Sng Y, Ong CK and Lai YF. Approaches to outpatient pharmacy automation: a systematic review. *Eur J Hosp Pharm* 2019; 26: 157-162. 2019/08/21. DOI: 10.1136/ejhpharm-2017-001424.
26. Mossialos E, Courtin E, Naci H, et al. From "retailers" to health care providers: Transforming the role of community pharmacists in chronic disease management. *Health Policy* 2015; 119: 628-639. DOI: 10.1016/j.healthpol.2015.02.007.
27. PharmData Ltd. PharmData, https://www.pharmdata.co.uk/ (2023, accessed 1st February 2023).

For peer review only

For peer review only - http://bmjopen.bmj.com/site/about/guidelines.xhtml

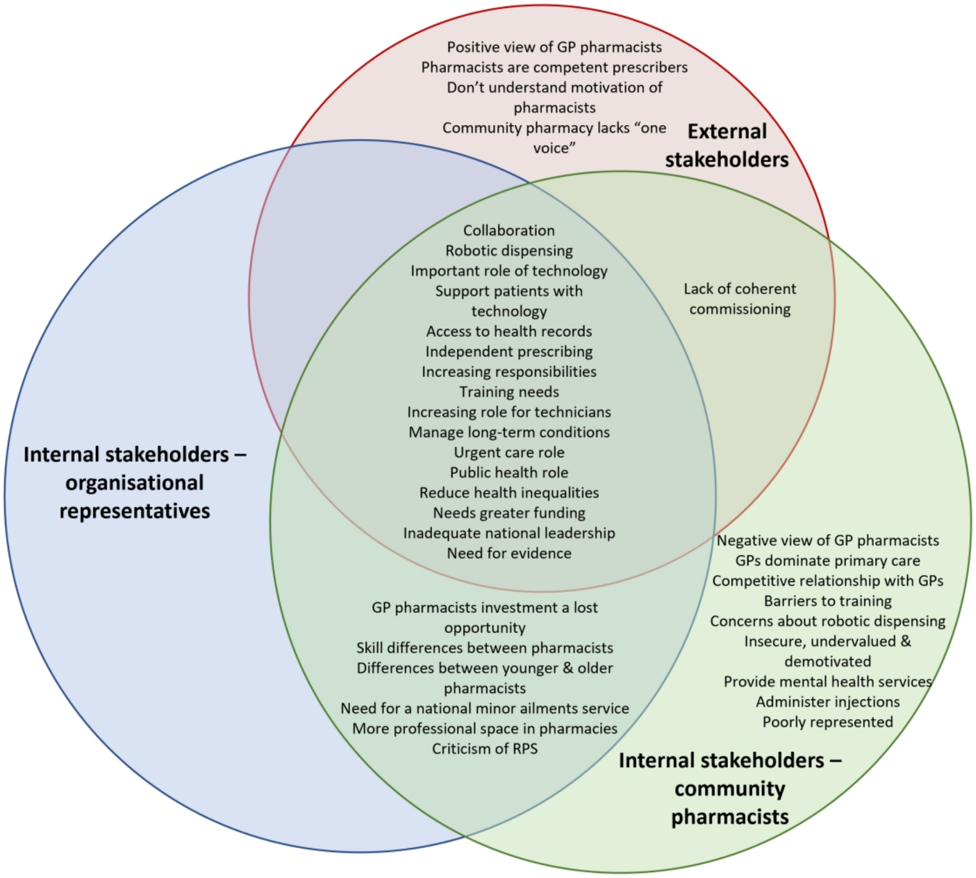

Figure 1: Areas of convergence and divergence in the views discussed by external stakeholders and internal stakeholders (both organisational representatives and community pharmacists).

532x470mm (130 x 130 DPI)

	Guide questions/description	Comment	Location in the manuscript (section)
**Domain 1: Research team and reflexivity**			
Personal Characteristics			
1. Interviewer/ facilitator	Which author/s conducted the interview or focus group?	Evgenia Paloumpi (EP) conducted the interviews.	2.2
2. Credentials	What were the researcher's credentials? E.g. PhD, MD	EP had MPharm and MRes degrees. MW, PO and MJ each had a PhD. EP, MW and MJ were all registered pharmacists.	N/A
3. Occupation	What was their occupation at the time of the study?	EP was a PhD student at Department of Pharmacy and Pharmacology, University of Bath.<br>MW, PO and MJ were EP's supervisors and full-time academics.	N/A
4. Gender	Was the researcher male or female?	EP and MW were female.<br>PO and MJ were male.	N/A
5. Experience and training	What experience or training did the researcher have?	EP had undertaken several courses related to advanced qualitative health research before the study was conducted. The other authors had extensive training and experience in qualitative health research.	N/A
Relationship with participants			
6. Relationship established	Was a relationship established prior to study commencement?	EP had previously met some participants at professional events such as conferences.	N/A
7. Participant knowledge of the interviewer	What did the participants know about the researcher? e.g. personal goals, reasons for doing the research	Potential participants were sent written information explaining the aims of the study and that it informed part of a PhD project.<br><br>Prior the interviews, EP introduced herself again as a PhD student and described the purpose of the project, before answering any questions that participants may have had.	2.1

For peer review only

8. Interviewer characteristics	What characteristics were reported about the interviewer/facilitator? e.g. Bias, assumptions, reasons and interests in the research topic	EP is a pharmacist with a particular interest in health policy.<br>MW is a pharmacist with a particular interest in community pharmacy research.<br>MJ is a pharmacist with experience of work as a community pharmacy locum and research interests in medicines safety and medicines information.<br>PO is a health policy researcher.	N/A
**Domain 2: Study design**			
Theoretical framework			
9. Methodological orientation and Theory	What methodological orientation was stated to underpin the study? e.g. grounded theory, discourse analysis, ethnography, phenomenology, content analysis	Underpinning ontology and epistemology were interpretivist. A reflexive thematic analysis using inductive approaches was applied. The Walt and Gilson policy framework was used to develop the topic guide.	2.3
Participant selection			
10. Sampling	How were participants selected? e.g. purposive, convenience, consecutive, snowball	Organisational representatives were selected using stakeholder mapping and snowballing. Community pharmacists were selected purposively.	2.1
11. Method of approach	How were participants approached? e.g. face-to-face, telephone, mail, email	Organisational representatives were contacted directly in writing and by telephone.<br><br>Community pharmacists were approached via advertisements in the Royal Pharmaceutical Society online community pharmacy forum, 48 local pharmaceutical committees, the researchers' networks and social media.<br><br>A study information sheet was emailed to these individuals who agreed to take part.	2.1
12. Sample size	How many participants were in the study?	Twenty-five	3.1

For peer review only

13. Nonparticipation	How many people refused to participate or dropped out? Reasons?	No interviewees were recruited from the following groups: public representatives; pharmacy and allied healthcare professionals (The Association of Independent Multiple Pharmacies, Boots Pharmacists Association, Royal College of General Practitioners, British Medical Association, Royal College of Nursing); commissioners and policymakers (Department of Health and Social Care, All-Party Pharmacy Group); and experts and collaborators (The King's Fund). The most common reasons for non-recruitment were either no response received or time restrictions of interviewees.	N/A
Setting			
14. Setting of data collection	Where was the data collected? e.g. home, clinic, workplace	Interviewees could determine whether they preferred to be interviewed face-to-face or by telephone. All face-to-face interviews took place in nonclinical professional area.<br><br>Five interviews were conducted in person at a location convenient to participants (e.g. participants' offices), 19 by telephone and one by Skype using videocall.	2.2
15. Presence of non-participants	Was anyone else present besides the participants and researchers?	No.	N/A
16. Description of sample	What are the important characteristics of the sample? e.g. demographic data, date	-	3.1 and Table S2 in this supplementary material
Data collection			
17. Interview guide	Were questions, prompts, guides provided by the authors? Was it pilot tested?	An interview topic guide (see below) was developed based on a recent policy review, previous research, supervisory team discussions and the Walt and Gilson policy framework (content, context, process and actors). The final interview guide included 11, broad, open-ended questions and probes to allow a flexible conversation with participants, with the aim of understanding their views on how	Below

For peer review only

		pharmaceutical care in the community would be provided in 2030. The topic guide and interviewer's technique were piloted interview with a stakeholder who was not included in the study as a participant. The pilot provided useful insights and feedback on interview conduct (e.g. ensuring availability for the interview duration before commencing it). The topic guide evolved progressively as interviews were carried out, to ensure that findings informed subsequent interviews. Questions 7, 8 and 9 were added to the topic guide as many participants referred to these topics during their interviews.	
18. Repeat interviews	Were repeat interviews carried out? If yes, how many?	No.	N/A
19. Audio/visual recording	Did the research use audio or visual recording to collect the data?	All interviews were audio-recorded (with permission) and later transcribed verbatim.	2.2
20. Field notes	Were field notes made during and/or after the interview or focus group?	Hand-written notes with key points and observations of the interview were also made by the researcher.	2.2
21. Duration	What was the duration of the interviews or focus group?	Interview duration varied from 35 to 75 minutes (mean = 60 minutes).	3.1
22. Data saturation	Was data saturation discussed?	Participants in this study were not recruited based on data saturation estimates as every stakeholder's view was considered useful for the future development of pharmaceutical care. Recruitment continued until all identified stakeholders had been interviewed or had declined.	2.1
23. Transcripts returned	Were transcripts returned to participants for comment and/or correction?	Transcripts were not returned to participants for comments due to time restrictions.	N/A
**Domain 3: Analysis and findings**			
Data analysis			

For peer review only

24. Number of data coders	How many data coders coded the data?	Coding was led by one researcher (EP), with regular discussion and agreement with the team.	2.3
25. Description of the coding tree	Did authors provide a description of the coding tree?		3.2-2.6
26. Derivation of themes	Were themes identified in advance or derived from the data?	This study used an inductive approach. The codes and the themes were informed by interview content.	2.3
27. Software	What software, if applicable, was used to manage the data?	NVivo12® software was used.	2.3
28. Participant checking	Did participants provide feedback on the findings?	No.	N/A
Reporting			
29. Quotations presented	Were participant quotations presented to illustrate the themes/findings? Was each quotation identified? e.g. participant number	Yes, quotations were presented and identified in a manner protecting participant confidentiality.	3.3-3.6
30. Data and findings consistent	Was there consistency between the data presented and the findings?	Yes, there was consistency between the data and the findings.	3.2-3.6
31. Clarity of major themes	Were major themes clearly presented in the findings?	Yes, major themes were clearly identified.	3.2-3.6
32. Clarity of minor themes	Is there a description of diverse cases or discussion of minor themes?	Yes, minor themes and diverse cases were clearly identified and related to major themes.	3.2-3.6

N/A; not applicable

Tong A, Sainsbury P, and Craig J. Consolidated criteria for reporting qualitative research (COREQ): a 32-item checklist for interviews and focus groups. *Int J Qual Health Care* 2007;19:349–57.

For peer review only

BMJ Open

Professional stakeholders' expectations for the future of community pharmacy practice in England: a qualitative study

Supplemental material

Evgenia Paloumpi[a], Piotr Ozieranski[b], Margaret C Watson[c] and Matthew D. Jones[a]

[a]Department of Life Sciences, University of Bath, Bath, England.

[b]Department of Social & Policy Sciences, University of Bath, Bath, England.

[c]Strathclyde Institute of Pharmacy and Biomedical Sciences, University of Strathclyde, Glasgow, Scotland.

For peer review only

Table S1: Consolidated criteria for reporting qualitative research (COREQ) 32-item checklist

	Guide questions/description	Comment	Location in the manuscript (section)
**Domain 1: Research team and reflexivity**			
Personal Characteristics			
1. Interviewer/ facilitator	Which author/s conducted the interview or focus group?	Evgenia Paloumpi (EP) conducted the interviews.	2.2
2. Credentials	What were the researcher's credentials? E.g. PhD, MD	EP had MPharm and MRes degrees. MW, PO and MJ each had a PhD. EP, MW and MJ were all registered pharmacists.	N/A
3. Occupation	What was their occupation at the time of the study?	EP was a PhD student at Department of Pharmacy and Pharmacology, University of Bath.<br>MW, PO and MJ were EP's supervisors and full-time academics.	N/A
4. Gender	Was the researcher male or female?	EP and MW were female.<br>PO and MJ were male.	N/A
5. Experience and training	What experience or training did the researcher have?	EP had undertaken several courses related to advanced qualitative health research before the study was conducted. The other authors had extensive training and experience in qualitative health research.	N/A
Relationship with participants			
6. Relationship established	Was a relationship established prior to study commencement?	EP had previously met some participants at professional events such as conferences.	N/A
7. Participant knowledge of the interviewer	What did the participants know about the researcher? e.g. personal goals, reasons for doing the research	Potential participants were sent written information explaining the aims of the study and that it informed part of a PhD project.<br><br>Prior the interviews, EP introduced herself again as a PhD student and described the purpose of the project, before answering any questions that participants may have had.	2.1

8. Interviewer characteristics	What characteristics were reported about the interviewer/facilitator? e.g. Bias, assumptions, reasons and interests in the research topic	EP is a pharmacist with a particular interest in health policy.<br>MW is a pharmacist with a particular interest in community pharmacy research.<br>MJ is a pharmacist with experience of work as a community pharmacy locum and research interests in medicines safety and medicines information.<br>PO is a health policy researcher.	N/A
**Domain 2: Study design**			
Theoretical framework			
9. Methodological orientation and Theory	What methodological orientation was stated to underpin the study? e.g. grounded theory, discourse analysis, ethnography, phenomenology, content analysis	Underpinning ontology and epistemology were interpretivist. A reflexive thematic analysis using inductive approaches was applied. The Walt and Gilson policy framework was used to develop the topic guide.	2.3
Participant selection			
10. Sampling	How were participants selected? e.g. purposive, convenience, consecutive, snowball	Organisational representatives were selected using stakeholder mapping and snowballing. Community pharmacists were selected purposively.	2.1
11. Method of approach	How were participants approached? e.g. face-to-face, telephone, mail, email	Organisational representatives were contacted directly in writing and by telephone.<br><br>Community pharmacists were approached via advertisements in the Royal Pharmaceutical Society online community pharmacy forum, 48 local pharmaceutical committees, the researchers' networks and social media.<br><br>A study information sheet was emailed to these individuals who agreed to take part.	2.1
12. Sample size	How many participants were in the study?	Twenty-five	3.1

13. Nonparticipation	How many people refused to participate or dropped out? Reasons?	No interviewees were recruited from the following groups: public representatives; pharmacy and allied healthcare professionals (The Association of Independent Multiple Pharmacies, Boots Pharmacists Association, Royal College of General Practitioners, British Medical Association, Royal College of Nursing); commissioners and policymakers (Department of Health and Social Care, All-Party Pharmacy Group); and experts and collaborators (The King's Fund). The most common reasons for non-recruitment were either no response received or time restrictions of interviewees.	N/A
Setting			
14. Setting of data collection	Where was the data collected? e.g. home, clinic, workplace	Interviewees could determine whether they preferred to be interviewed face-to-face or by telephone. All face-to-face interviews took place in nonclinical professional area.<br><br>Five interviews were conducted in person at a location convenient to participants (e.g. participants' offices), 19 by telephone and one by Skype using videocall.	2.2
15. Presence of non-participants	Was anyone else present besides the participants and researchers?	No.	N/A
16. Description of sample	What are the important characteristics of the sample? e.g. demographic data, date	-	3.1 and Table S2 in this supplementary material
Data collection			
17. Interview guide	Were questions, prompts, guides provided by the authors? Was it pilot tested?	An interview topic guide (see below) was developed based on a recent policy review, previous research, supervisory team discussions and the Walt and Gilson policy framework (content, context, process and actors). The final interview guide included 11, broad, open-ended questions and probes to allow a flexible conversation with participants, with the aim of understanding their views on how	Below

For peer review only

		pharmaceutical care in the community would be provided in 2030. The topic guide and interviewer's technique were piloted interview with a stakeholder who was not included in the study as a participant. The pilot provided useful insights and feedback on interview conduct (e.g. ensuring availability for the interview duration before commencing it). The topic guide evolved progressively as interviews were carried out, to ensure that findings informed subsequent interviews. Questions 7, 8 and 9 were added to the topic guide as many participants referred to these topics during their interviews.	
18. Repeat interviews	Were repeat interviews carried out? If yes, how many?	No.	N/A
19. Audio/visual recording	Did the research use audio or visual recording to collect the data?	All interviews were audio-recorded (with permission) and later transcribed verbatim.	2.2
20. Field notes	Were field notes made during and/or after the interview or focus group?	Hand-written notes with key points and observations of the interview were also made by the researcher.	2.2
21. Duration	What was the duration of the interviews or focus group?	Interview duration varied from 35 to 75 minutes (mean = 60 minutes).	3.1
22. Data saturation	Was data saturation discussed?	Participants in this study were not recruited based on data saturation estimates as every stakeholder's view was considered useful for the future development of pharmaceutical care. Recruitment continued until all identified stakeholders had been interviewed or had declined.	2.1
23. Transcripts returned	Were transcripts returned to participants for comment and/or correction?	Transcripts were not returned to participants for comments due to time restrictions.	N/A
**Domain 3: Analysis and findings**			
Data analysis			

For peer review only

24. Number of data coders	How many data coders coded the data?	Coding was led by one researcher (EP), with regular discussion and agreement with the team.	2.3
25. Description of the coding tree	Did authors provide a description of the coding tree?		3.2-2.6
26. Derivation of themes	Were themes identified in advance or derived from the data?	This study used an inductive approach. The codes and the themes were informed by interview content.	2.3
27. Software	What software, if applicable, was used to manage the data?	NVivo12® software was used.	2.3
28. Participant checking	Did participants provide feedback on the findings?	No.	N/A
Reporting			
29. Quotations presented	Were participant quotations presented to illustrate the themes/findings? Was each quotation identified? e.g. participant number	Yes, quotations were presented and identified in a manner protecting participant confidentiality.	3.3-3.6
30. Data and findings consistent	Was there consistency between the data presented and the findings?	Yes, there was consistency between the data and the findings.	3.2-3.6
31. Clarity of major themes	Were major themes clearly presented in the findings?	Yes, major themes were clearly identified.	3.2-3.6
32. Clarity of minor themes	Is there a description of diverse cases or discussion of minor themes?	Yes, minor themes and diverse cases were clearly identified and related to major themes.	3.2-3.6

N/A; not applicable

Tong A, Sainsbury P, and Craig J. Consolidated criteria for reporting qualitative research (COREQ): a 32-item checklist for interviews and focus groups. *Int J Qual Health Care* 2007;19:349–57.

For peer review only

Interview topic guide

Opening

- Researcher introduction and brief overview explaining purpose of the study and describing the term "pharmaceutical care in the community" used for the study

  "My name is Evina and I am a PhD student from University of Bath. As you already know, my research aims to develop a more evidence-based vision for the future of community pharmacy services in England. We are having this conversation to discuss your views on how pharmaceutical care will be provided in the community in the year 2030. And when I say pharmaceutical care, I would describe this discipline as helping patients use and manage medicines effectively, but also providing wider interventions that could create an impact on their quality of life."

- Check participant has read Participant Information Sheet (provide a copy and time to read if they haven't)
- Ask participant if they have any questions about the study
- Explain to the participants about the informed consent
- Ask how much time the participant has available and plan questions – "*Our interview is not expected to last more than one hour but if you have other commitments, we can finish earlier than that."*
- Secure consent
- Start recording the interview

Main Discussion

Probes will be used flexibly, in response to participant's answers.

1. How would you describe your [members'] role in relation to this sort of pharmaceutical care in the community?	**Role**:
2. In your view, in what ways might pharmaceutical care in the community be provided in the year 2030?<br><br>*Probe common topics from policy review:*<br>• *urgent and emergency care*<br>• *mental health (dementia friend)*<br>• *closer collaborations with primary care professionals*<br>• *long-term conditions*<br>• *patient-centred care*<br>• *new models of care*	**Ways**:
3. Which of these developments are more like likely to happen and why?	**More likely to happen & why**:

For peer review only

Probe common drivers from policy review:<br>• *unnecessary workload in general practice*<br>• *increased service demands*<br>• *funding allocation*<br>• *increased hospital admissions*	
4. We have already discussed the different ways you think that pharmaceutical care will be provided in the year 2030. Suppose that you had an opportunity to create the ideal model of pharmaceutical care in the community, but without increasing the total cost. What would this ideal model include?	**Ideal model:**
5. [If answer to Q4 different to Q3] Your ideal model is different to what you think will actually happen. Why do you think your ideal model is unlikely to come about?	**Why expectation different to ideal:**
6. What would make your ideal model more likely to come about?<br><br>*Probe common facilitators and barriers for Q5 and Q6:*<br>• *individual behaviour*<br>• *utilising technology*<br>• *supporting workforce*<br>• *providing evidence*<br>• *facilitate commissioning*	**What would make ideal more likely:**
7. What are the current barriers to pharmaceutical care provision in the community?	**Barriers:**
8. What are your views on the role of pharmacy technicians in pharmaceutical care?	**Technicians:**
9. What are your views on community pharmacy mergers and closures due to financial constraints?	**Mergers and closures:**
10. Do you feel there is anything else you would like to add?	**Additional:**

For peer review only

Closing

- Summarise main points of the discussion
- One last question from me “What other organisation or individual would you consider influential for the future of pharmaceutical care in the community?
- Ask if they would like a copy of the results to be sent to them
- Thank participants for their time

For peer review only

Table S2: Interviewee characteristics

Type of interviewee	Number of interviewees	Organisations with whom interviewees were linked
**External stakeholders**	7<br>(4 female, 3 male)	NHS England<br>NHS Clinical Commissioning<br>General practice<br>National Association of Primary Care<br>New NHS Alliance<br>Academics from UK universities<br>Pharmacy-related media
**Internal stakeholders – organisational representatives**	8<br>(5 female, 3 male)	Royal Pharmaceutical Society<br>Pharmaceutical Services Negotiating Committee<br>National Pharmacy Association<br>Company Chemists' Association<br>Pharmacists' Defence Association[1]<br>Association of Pharmacy Technicians UK<br>Pharmaceutical industry
**Internal stakeholders – community pharmacists[2,3]**	10<br>(2 female, 8 male)	Independent community pharmacy (n=7)<br>Multiple community pharmacy (n=7)<br>Locum pharmacist (n=4)<br>Local pharmaceutical committees (n=2)<br>Pharmacy education bodies (n=2)

[1]Two interviewees linked to the Pharmacists' Defence Association were interviewed

[2]A multiple community pharmacy contractor was defined as consisting of six pharmacies or more; contractors with five or fewer pharmacies were regarded as independent.

[3]Some community pharmacists were linked to more than one type of organisation.

For peer review only

Table S3: Characteristics of interviewees from all backgrounds

ID number	Organisational stakeholders			Community pharmacist internal stakeholders				
	**External**	**Internal**	**Gender**	**Independent community pharmacy[1]**	**Multiple community pharmacy [1]**	**Locum**	**Gender**	**Region in England**
**1**				✓	✓	✓	Male	North
**2**		✓	Male					
**3**		✓	Female					
**4**		✓	Male					
**5**		✓	Male					
**6**				✓	✓	✓	Female	Midlands and East
**7**				✓			Male	North
**8**	✓		Female					
**9**		✓	Female					
**10**				✓			Male	Midlands and East
**11**		✓	Female					
**12**	✓		Male					
**13**				✓	✓		Male	North
**14**				✓			Male	South
**15**	✓		Male					
**16**	✓		Female					
**17**	✓		Female					
**18**		✓	Female					
**19**					✓		Male	South
**20**	✓		Male					
**21**					✓	✓	Female	South
**22**				✓	✓	✓	Male	North, Midlands and East
**23**		✓	Female					
**24**					✓		Male	North
**25**	✓		Female					
**TOTAL**	**7**	**8**	**Male (n=6) Female (n=9)**	**7**	**7**	**4**	**Male (n=8) Female (n=2)**	-

[1]A multiple community pharmacy contractor was defined as consisting of 6 pharmacies or more; contractors with 5 or fewer pharmacies were regarded as independent (General Pharmaceutical Services in England 2008/09 - 2018/19, NHS Digital).

For peer review only

BMJ Open

Professional stakeholders' expectations for the future of community pharmacy practice in England: a qualitative study

Journal:	*BMJ Open*
Manuscript ID	bmjopen-2023-075069.R1
Article Type:	Original research
Date Submitted by the Author:	19-Jul-2023

Complete List of Authors:	Paloumpi, Evgenia; University of Bath, Department of Life Sciences<br>Ozieranski, Piotr; University of Bath, Department of Social & Policy Sciences<br>Watson, Margaret; University of Strathclyde<br>Jones, Matthew; University of Bath, Department of Life Sciences

**Primary Subject Heading**:	General practice / Family practice
Secondary Subject Heading:	Health policy, Health services research, Pharmacology and therapeutics, Qualitative research
Keywords:	Primary Care < Primary Health Care, Health policy < HEALTH SERVICES ADMINISTRATION & MANAGEMENT, Organisation of health services < HEALTH SERVICES ADMINISTRATION & MANAGEMENT, PUBLIC HEALTH, QUALITATIVE RESEARCH

SCHOLARONE™
Manuscripts

I, the Submitting Author has the right to grant and does grant on behalf of all authors of the Work (as defined in the below author licence), an exclusive licence and/or a non-exclusive licence for contributions from authors who are: i) UK Crown employees; ii) where BMJ has agreed a CC-BY licence shall apply, and/or iii) in accordance with the terms applicable for US Federal Government officers or employees acting as part of their official duties; on a worldwide, perpetual, irrevocable, royalty-free basis to BMJ Publishing Group Ltd ("BMJ") its licensees and where the relevant Journal is co-owned by BMJ to the co-owners of the Journal, to publish the Work in this journal and any other BMJ products and to exploit all rights, as set out in our licence.

The Submitting Author accepts and understands that any supply made under these terms is made by BMJ to the Submitting Author unless you are acting as an employee on behalf of your employer or a postgraduate student of an affiliated institution which is paying any applicable article publishing charge ("APC") for Open Access articles. Where the Submitting Author wishes to make the Work available on an Open Access basis (and intends to pay the relevant APC), the terms of reuse of such Open Access shall be governed by a Creative Commons licence – details of these licences and which Creative Commons licence will apply to this Work are set out in our licence referred to above.

Other than as permitted in any relevant BMJ Author's Self Archiving Policies, I confirm this Work has not been accepted for publication elsewhere, is not being considered for publication elsewhere and does not duplicate material already published. I confirm all authors consent to publication of this Work and authorise the granting of this licence.

For peer review only

Professional stakeholders' expectations for the future of community pharmacy practice in England: a qualitative study

Evgenia Paloumpi[a]*, Piotr Ozieranski[b], Margaret C Watson[c] and Matthew D. Jones[d] (corresponding author)

[a]Department of Life Sciences, University of Bath, Bath, England. evina.paloumpi@gmail.com

[b]Department of Social & Policy Sciences, University of Bath, Bath, England. P.Ozieranski@bath.ac.uk

[c]Strathclyde Institute of Pharmacy and Biomedical Sciences, University of Strathclyde, Glasgow, Scotland. margaret.watson@strath.ac.uk

[d]Department of Life Sciences, University of Bath, Bath, England. M.D.Jones@bath.ac.uk, telephone: +44 1225 383829

*The views expressed in this paper are purely those of the author. They do not necessarily reflect the views or official positions of the European Commission and the ERC Executive Agency.

For peer review only - http://bmjopen.bmj.com/site/about/guidelines.xhtml

Abstract

Objectives: To explore the views of professional stakeholders on the future of community pharmacy services in England. Specific objectives related to expectations of how community pharmacy services will be provided by 2030 and factors that will influence this.

Design: Qualitative, using semi-structured interviews in person or via telephone/Skype. The topic guide was informed by a recent policy review that used the Walt and Gilson policy framework. Transcripts were analysed using inductive thematic analysis.

Setting: England

Participants: External stakeholders were representatives of non-pharmacy organisations, including policymakers, commissioners and representatives of healthcare professions. Internal stakeholders were community pharmacists or pharmacy organisations representatives. Interviewees were identified using stakeholder mapping

Results: In total, 25 interviews were completed (7 external stakeholders and 18 internal stakeholders, of which 10 were community pharmacists). Community pharmacy was recognised as having a key role in expanding health system capacity *("…pharmacy is the obvious person to take on those roles…"*), particularly for long-term condition management (e.g. adherence, reducing polypharmacy, monitoring), urgent care (e.g. minor illnesses) and public health (including mental health). For these contributions to be developed and optimised, greater integration and collaboration with general practices will be needed (*"…there is no room for isolationism in pharmacy anymore…"*), as well as use of technology in a patient-centred way and full access to health records. These changes will require workforce development together with appropriate commissioning and contractual

For peer review only - http://bmjopen.bmj.com/site/about/guidelines.xhtml

arrangements. Community pharmacy is currently undervalued (*"…the complete misunderstanding by senior Government officials is very scary"*) and recent investment in general practice pharmacists rather than community pharmacy was seen as a missed opportunity.

Conclusions: Community pharmacy as a sector could and should be developed to increase health service capacity to address its current challenges. Numerous modifications are required from a range of stakeholders to create the environment in which these changes can occur.

Strengths and limitations of this study

- The key strength of this study is its examination of the development of community pharmacy services in England in their entirety (rather than individual services) from the perspective of a wide variety of systematically selected interviewees, making it more representative and generalisable than some previous studies.
- The inability to recruit stakeholders from the Government and organisations that represent the public limits the breadth of perspectives included.
- The addition of pharmacists working in other sectors (e.g. GP pharmacists) could have added useful insights.

For peer review only

For peer review only - http://bmjopen.bmj.com/site/about/guidelines.xhtml

Funding statement

This work was supported by Sigma Pharmaceuticals via the Bharat Shah PhD Scholarship (award/grant no: NA) and the Harold and Marjorie Moss Charitable Trust Fund (award/grant no: NA).

Competing interests

None declared.

Data sharing statement

Not applicable due to lack of participant consent.

Word count

6064 words

Keywords

Community pharmacy services; England; stakeholder participation.

For peer review only

1.0 Introduction

The National Health Service (NHS) in England (the largest and most populous part of the United Kingdom) is currently facing unprecedented work pressures. In primary care, increased patient demand combined with a reduction in the number of general practitioners (GPs), poor morale and decreased real-terms funding per capita has led many to talk of a system in "crisis" (1). The secondary care sector is facing similar challenges (2).

Community pharmacies are one of four fundamental sectors of primary care in England (3). They are easily accessible premises where qualified healthcare professionals supply medicines and provide medicines-related and public health services. There is evidence of the effectiveness of community pharmacy services to manage long-term conditions (4), minor ailments (5) and promote public health (6) from systematic reviews and quantitative primary research. Greater use of community pharmacy has therefore been identified as an important way to address current NHS challenges (7). However, despite the substantial contribution that community pharmacies make to the NHS throughout England, particularly during the Covid-19 pandemic, the sector is experiencing sustained underfunding (8), increased rates of closure (9) and growing competition from online pharmacies (10).

In addition, since 2015 there has been a large increase in the number of 'general practice pharmacists' in England (11). This is a distinct role to that of community pharmacists, responsible for various tasks in general practices, such as structured medication reviews and medicines reconciliation. The impact of GP pharmacists on community pharmacy has not been widely explored.

For peer review only - http://bmjopen.bmj.com/site/about/guidelines.xhtml

A tipping point is being reached for community pharmacy in England, with an urgent need for a national vision and strategy (12). A multi-stakeholder perspective combined with a system-wide approach is needed to ensure that strategic developments reflect not only need but capabilities. There has been no recent exploration of stakeholders' visions for the future of the community pharmacy and earlier consultations have focused on singular activities.

This study explored the views of health-sector stakeholders on the short-to-midterm (10-year) future of community pharmacy in England. Specific objectives were to explore:

- Expectations of how community pharmacy services will be provided by 2030;
- Factors that will influence future service development and delivery;
- Opinions of how community pharmacy services should ideally be provided;
- Facilitators and barriers to the development these ideal community pharmacy services.

2.0 Methods

A qualitative interview design was chosen to allow in-depth exploration of stakeholders' views. The Research Ethics Approval Committee for Health (REACH) of the University of Bath (reference EP 17/18 204) approved the study in August 2018. Reporting is in accordance with the Consolidated Criteria for Reporting Qualitative Research (Table S1, supplementary material).

For peer review only - http://bmjopen.bmj.com/site/about/guidelines.xhtml

2.1. Interviewees and recruitment

Both internal and external stakeholders were recruited. External stakeholders were representatives of non-pharmacy organisations (Table S2, supplementary material). Internal stakeholders were either current community pharmacists or representatives of pharmacy organisations. Internal and external organisational representatives were recruited by stakeholder mapping and direct invitation, whereas community pharmacists were recruited via advertisement. Interviewees gave written or audio informed consent.

Stakeholder mapping was used to systematically identify and invite a broad range internal and external organisational representatives (such as policymakers, commissioners and representatives of patients or healthcare professionals) to participate (13). An initial list of stakeholders was constructed using a policy review (14) and research team expertise (Table S2, supplementary material). A snowballing approach was also adopted to supplement any underrepresented stakeholder categories. Stakeholder profiles (career history, position, interest and influence) were created using publicly accessible information. These were used to select stakeholders to invite to interview. Up to five stakeholders were identified and ranked within each organisation. The highest ranked stakeholder was sent a written invitation, with telephone follow-up if needed. If a stakeholder declined, the next ranked individual from their organisation was invited. This process continued until a representative of every organisation been interviewed or all the identified stakeholders from an organisation had declined their invitation.

For peer review only - http://bmjopen.bmj.com/site/about/guidelines.xhtml

Community pharmacists were eligible for inclusion if they worked regularly in a community pharmacy in England and were identified using purposive sampling, to obtain a diverse sample based on a sampling matrix including type of community pharmacy, locum status (as these pharmacists work in multiple pharmacies and so may have different perspectives), gender and geographical region. The study was advertised to community pharmacists via the Royal Pharmaceutical Society's (i.e. the professional body for pharmacy) online forums, local pharmaceutical committees, the research team's networks and social media. Every eligible person who responded to these advertisements was interviewed.

2.2. Data collection

Interviews were carried out by one trained researcher (EP) face-to-face, by telephone or Skype, to provide flexibility and increase recruitment (15). A topic guide (supplementary material) was developed based on a recent policy review (14) that employed the Walt and Gilson policy framework (16) and discussion within the research team. One pilot interview was conducted. Interviews were audio-recorded, transcribed verbatim and checked for accuracy and anonymity. The interviewer made notes of key points and observations from the interview.

2.3. Data analysis

Interview transcripts were analysed using inductive thematic analysis, which is suitable for applied health research aiming to inform the development of policy (17). Analysis was iterative, based on a six-phase process: data familiarisation; generating initial codes; developing themes; reviewing themes; defining and naming themes; writing the analysis

For peer review only - http://bmjopen.bmj.com/site/about/guidelines.xhtml

(17). Initial manual coding was then interpreted to generate initial themes and subthemes using NVivo v.12 software. Themes were iteratively refined for cohesiveness using a thematic map, before the final themes were named and defined. These processes were led by one researcher (EP), with regular discussion and agreement with the team.

2.4 Patient and public involvement statement

Patients and the public were not involved in this study. A separate study examining the perspectives of members of the public has been completed and will be reported (18).

3.0 Results

3.1 Interviewee characteristics

In total, 46 stakeholders were invited and 25 interviews were completed between November 2018 and April 2019. There were seven external stakeholders from seven organisations and eighteen internal stakeholders. Of these, eight were organisational representatives (from seven organisations) and ten were community pharmacists (Tables S3 and S4, supplementary material). Stakeholders representing three patient organisations, two state policy making organisations, two pharmacy representative organisations and three organisations representing other healthcare professions were invited, but did not participate. Interview duration varied from 35 to 75 minutes.

For peer review only - http://bmjopen.bmj.com/site/about/guidelines.xhtml

For peer review only

3.2 Overview of themes

Four themes with 11 sub-themes were generated from the analysis (Table 1). Each of these will be discussed in order of dominance.

Table 1: Themes and sub-themes generated from the analysis.

Theme	Sub-theme
Creating collaborations for wider pharmacy services provision from community pharmacy	Collaboration: expected, ideal and a facilitator
	Existing relationships with general practices
Integrating new technology into pharmacy services	Technology expected to change community pharmacy practice
	Making technology work for patients
	Information-sharing technology
Building workforce competency	Increasing responsibilities with a decreasing workforce
	Enhancing the skills of the workforce
	Role of pharmacy technicians
Future community pharmacy services	Community pharmacists' role in managing chronic conditions, urgent care and prevention
	Opportunities for further community pharmacy development
	Creating the environment for future developments

There were large areas of convergence in the views discussed by external stakeholders and internal stakeholders (both organisational representatives and community pharmacists) (Figure 1). In particular, they related to community pharmacy services, technology and the workforce. There was also considerable divergence with only community pharmacists discussing how the sector felt insecure and undervalued and expressing concerns about technology and collaborative relationships.

For peer review only - http://bmjopen.bmj.com/site/about/guidelines.xhtml

[Insert Figure 1]

Figure 1: Areas of convergence and divergence in the views discussed by external stakeholders and internal stakeholders (both organisational representatives and community pharmacists).

3.3 Theme 1: Collaboration for wider pharmacy services provision from community pharmacy

Subtheme: Collaboration: expected, ideal and a facilitator

> *"There is a coming together of health and social care…and I think pharmacy has to be in that tent…I don't see any future…for a pharmacy as a kind of standalone… there is no room for isolationism in pharmacy anymore it has to be seen as part of a wider team."*
>
> Interviewee ID12 – external stakeholder (GP)

Interviewees expected future community pharmacy services to be provided collaboratively with other healthcare professionals and voluntary organisations. They envisaged more integration between professions with greater awareness of other professionals' skills and an understanding that services could be provided differently. Interviewees expected community pharmacists to work within local groups of general practices (known as primary care networks in England) and viewed current involvement as an encouraging step towards integration into primary care. Interviewees also described community pharmacists' strategic involvement in local and national structures as part of an ideal model of care. They

For peer review only - http://bmjopen.bmj.com/site/about/guidelines.xhtml

emphasized that a community pharmacy culture change that promotes collaborative working would be crucial for patient care and the sector's survival.

Subtheme: Existing relationships with general practices

> *"…patients speak the language of medicines in terms of 'the blue ones' or 'the pink ones'… community pharmacists…straddle a divide between how patients think and talk and how the system thinks and talks…I deal with medicines every day of the week but somebody who is sat at their desk [GP pharmacist] wouldn't have a clue where they start with that."*
>
> Interviewee ID14 – community pharmacist

There were contrasting opinions about the role of GP pharmacists in supporting community pharmacy services: external stakeholders viewed GP pharmacists as facilitators, but community pharmacists referred to them as a barrier. Internal stakeholders were disappointed that NHS England had invested mostly in GP pharmacists rather than the existing community pharmacy workforce, describing this as a missed opportunity. There was concern over a division between 'clinical' GP pharmacists and 'community' pharmacists, and some thought that community pharmacists were better placed to support patients, due to better practical knowledge of patients' current medicines and how they are using them.

For peer review only - http://bmjopen.bmj.com/site/about/guidelines.xhtml

"You only need to look at the debacle with the flu service…both GPs and pharmacists think they are in competition with each other and rather than working collaboratively…for the whole population..."

Interviewee ID10 – community pharmacist

Many community pharmacists described competitive *"them and us"* (Interviewee ID20) relationships with GPs, resulting from conflicting financial incentives (e.g. competition between community pharmacies and GP practices to vaccinate the same pool of patients against influenza) and poor understanding of pharmacists' skills (e.g. a reluctance by some GPs to share health records due to the view that pharmacists are not trained to protect confidential information). However, some believed that GPs valued community pharmacists and were optimistic about future relationships.

Community pharmacists perceived GPs as dominating primary care, mentioning *"lack of parity"* (Interviewee ID14) in the distribution of services and highlighting the need to align the medical and pharmaceutical NHS contracts. Overall, the GP contract and workload pressures in general practice were the most influential factors driving pharmacy service development, as the capabilities of community pharmacy were only discussed by a minority of interviewees.

3.4 Theme 2: Integrating technology into pharmacy services

For peer review only - http://bmjopen.bmj.com/site/about/guidelines.xhtml

Subtheme: Technology expected to change community pharmacy practice

> *"If we can use IT robotics just to take care of certain aspects which are more tedious and laborious…the pharmacists will have much more time to devote to patient care."*
>
> Interviewee ID15 – external stakeholder

New technology was seen as integral to future pharmacy services and healthcare in general. This was attributed to patient expectations, the entry of technological companies into the medicines market and the Government's intentions.

Interviewees described a 'hub and spoke' model, with automated dispensing of medicines in a central location, away from community pharmacy premises. This was expected and perceived as ideal, and was usually viewed as enabling a change from a medicines supply model to one based on an enhanced clinical role. However, community pharmacists expressed concerns about delays to patient care and increased costs.

Home deliveries of medicines and increasing use of online pharmacies were commonly connected and were expected to increase, driven by convenience. Use of artificial intelligence to support patient decision-making, accurate prescribing and to reduce dispensing errors was also discussed speculatively, mainly by external stakeholders.

For peer review only

For peer review only - http://bmjopen.bmj.com/site/about/guidelines.xhtml

"… by 2030 Amazon could be delivering peoples' prescriptions door-to-door so where does pharmacy fit, if pharmacy hasn't got a more defined and a more developed clinical care role then, where is pharmacy?"

Interviewee ID12 – external stakeholder

Many highlighted the impact that these developments might have on the role of community pharmacists. Due to the risk of large wholesale companies dominating the medicines supply market, it will be necessary to establish a role based on supporting patients. A systematised, transactional and supply-only model for community pharmacy lacking knowledge of regular customers would have adverse consequences for patients.

Subtheme: Making technology work for patients

"…wearable technology…clinicians to deal remotely with the way that you are feeling…the fact that you are unwell can be detected at distance and then dealt with either at distance or…face-to-face..."

Interviewee ID4 – internal stakeholder

"But it's how patients use that medicine and how they become effective with the medicine that is going to require the clinical expertise of the community pharmacist and that clinical expertise has to be done really face-to-face so that you can see the patient more effectively."

For peer review only - http://bmjopen.bmj.com/site/about/guidelines.xhtml

Interviewee ID10 – community pharmacist

All interviewees described the growing role that healthcare applications (apps) and devices would have in pharmacy services, expecting transformed patient interaction for the supply of medicines, diagnosis and review of patient data. Future patients will require community pharmacists to understand and assist with these sources of information. Interviewees expected increased use of online consultations driven by patient expectations and preference but highlighted the importance of maintaining face-to-face contact with patients. Community pharmacists expressed concerns that face-to-face consultations were more effective. A 'mixed model' of digital and face-to-face communication was seen as ideal.

> *"…we have to be careful we don't generate technology poverty, so we don't want to exclude a sector of the population."*

Interviewee ID23 – internal stakeholder

Interviewees were cautious about how technology might exclude older generations. Community pharmacists were considered fundamental for preventing such inequalities and promoting familiarity with technology.

Subtheme: Information-sharing technology

For peer review only - http://bmjopen.bmj.com/site/about/guidelines.xhtml

"... I think once that link [read and write access to health records] is…established…in terms of what we can then do…it opens it up…anything and everything really."

Interviewee ID21 – community pharmacist

All interviewees referred to community pharmacist access to health records as a crucial facilitator for pharmacy services and for some community pharmacists this was ideal. It would allow seamless care, enable community pharmacists to manage long-term conditions more effectively and relieve GP workload. Current read-only access to Summary Care Records (an electronic summary of patients' key clinical information) was beneficial, but the ability to add information is required. Some interviewees thought this would enable community pharmacists to communicate medication changes, thus ensuring their advice is followed and providing GPs with an accurate picture of their interventions.

"The personalised care and the genomics is something that…pharmacists…need to make themselves really familiar with because that is the future..."

Interviewee ID18 – internal stakeholder

Interviewees expected new sources of patient data from developments such as pharmacogenomics. Professionals will need to understand and interpret these data, for which community pharmacists should be prepared.

3.5 Theme 3: Building workforce competence

For peer review only - http://bmjopen.bmj.com/site/about/guidelines.xhtml

Subtheme: Increasing responsibilities with a decreasing workforce

> *"The current Government agenda in relation to the closure of high street pharmacy, …I think that was scary…I have had a very senior person say to me we need to close these pharmacies because all they do is sell shampoo. I think the complete misunderstanding by senior Government officials is very scary."*
>
> Interviewee ID8 – external stakeholder

Most community pharmacists expressed insecurity about their future and felt their value was not recognised due to funding cuts and policies aiming to reduce the number of pharmacies. Lack of career development and incentives for community pharmacists was another factor contributing to a declining workforce. Community pharmacists referred to a tired, disillusioned and demotivated workforce working in tough conditions, warning that many might choose alternative careers (e.g. GP pharmacy), thus further reducing the workforce.

Nevertheless, interviewees also expected that community pharmacy responsibilities will increase, referring to community pharmacies becoming an increasingly clinical environment with a reduced medicines supply function. Some community pharmacists expected a transfer of services from GP practices. Therefore, interviewees envisaged that in 2030 there would be more than one pharmacist per pharmacy. For some, this was ideal, providing time for patient interaction and clinical roles.

For peer review only - http://bmjopen.bmj.com/site/about/guidelines.xhtml

Subtheme: Enhancing the skills of the workforce

> *"It is a no brainer to me that all pharmacists should be independent prescribers as an absolute pre-requisite…I think the independent prescribing as it currently stands is patchy it's not many community pharmacists doing it and it is not joined up, it is not really getting us anywhere."*
>
> Interviewee ID12 – external stakeholder

> *"I think at the moment the training for pharmacists working in community pharmacy is very piecemeal and there is…the problem of…getting released from work..."*
>
> Interviewee ID3 – internal stakeholder

It was clear that more community pharmacists should become independent prescribers with appropriate funding and training support. Prescribing was a facilitator that would allow community pharmacists to improve long-term condition management, run clinics for specific diseases and release more GP time for complex cases. External stakeholders supported the competence of pharmacists for this role.

Interviewees referred to the need to train the community pharmacy workforce for such responsibilities, including physical examinations, clinical assessment, consultation skills and data management. Many internal stakeholders believed there were skill differences in these areas between generations of pharmacists. Training alongside other healthcare

For peer review only - http://bmjopen.bmj.com/site/about/guidelines.xhtml

professionals, particularly doctors, will improve mutual understanding. Most community pharmacists described significant barriers to training, including time, funding and accessibility. They believed government expectations were unrealistic in the absence of protected time or funding, highlighting the need for more resources.

> *"Some pharmacists are their own worst enemy…a lot of them want to hold onto the dispensing process…they're quite happy with how things are..."*
>
> Interviewee ID1 – community pharmacist

Interviewees also referred to the sector's resistance to change as one of the biggest barriers to pharmacy services, especially those who might prefer the current situation or be reluctant to risk change to a profitable business. Some stakeholders believed that the businesses that were unwilling to change would suffer. Consequently, some external stakeholders highlighted the importance of understanding community pharmacists' values and motivations. Some internal stakeholders perceived younger pharmacists as advocating for a clinical role, with the contrasting dispensing-focus of some older pharmacist's seen as inconsiderate of their legacy to the future generations.

For peer review only

For peer review only - http://bmjopen.bmj.com/site/about/guidelines.xhtml

Subtheme: Role of pharmacy technicians

> *"…there are massively differing standards of technicians. There are some that I would trust to run a pharmacy with my life and there are others that I would not let near a pharmacy with a bargepole…"*
>
> Interviewee ID21 – community pharmacist

Interviewees recognised pharmacy technicians as underused and suggested making greater use of their skills. A variety of roles were suggested, such as enhancing their role in medication supply to increase cost-effectiveness and release more clinical time for pharmacists. Additional roles included simple consultations, smoking cessation, weight management, influenza vaccination and domiciliary visits. Training will be essential, considering the existing variability in the skills of pharmacy technicians described by some community pharmacists. However, funding for such training was a concern for some community pharmacists, as recent funding cuts had impacted on investment in technician development.

For peer review only - http://bmjopen.bmj.com/site/about/guidelines.xhtml

3.6 Theme 4: Future community pharmacy services

Subtheme: Community pharmacists' role in managing long-term conditions, urgent care and prevention

> *"These [long-term conditions]…cannot be dealt with by general practice alone and therefore pharmacy is the obvious person to come on and take on those roles so I can see that aspect growing significantly."*
>
> Interviewee ID20 – external stakeholder

Interviewees expected that community pharmacists' role in the management of long-term conditions would be enhanced in the future, to reduce medicine-related hospital admissions and release GP time. Community pharmacist support of medication adherence was already beneficial and there are opportunities for improving medicines safety, polypharmacy, monitoring and screening services. Drivers for these developments included current success, an existing team of capable staff, and rising multimorbidity, life expectancy and polypharmacy. Some community pharmacists viewed a more prominent role in long-term condition management as ideal, envisaging a future where they would be adequately resourced to monitor and manage medicines for long-term conditions such as asthma, diabetes and cardiovascular disease. However, some interviewees did not foresee further developments for community pharmacy in long-term condition management, with one warning of a *"difficult journey"* (interviewee ID12 – external stakeholder) requiring collaborations with other healthcare professionals that is difficult for pharmacists who are

For peer review only - http://bmjopen.bmj.com/site/about/guidelines.xhtml

“tied to a pharmacy” (interviewee ID9 – internal stakeholder) with limited capacity to take on additional roles and access training. This sub-theme therefore links to others related to collaboration, the potential of technology to change pharmacy practice and workforce competence.

> *“There would need to be a change in the…training that a pharmacist gets where it becomes more of a hybrid between being a pharmacist and being a doctor-physician’s assistant…without that additional clinic support it’s difficult to see how emergency services offered through community pharmacies would grow much beyond the current minor ailment-type model.”*
>
> Interviewee ID20 – external stakeholder

Further developments in urgent and emergency care were also expected. Interviewees identified this as an area where the community pharmacist’s role has progressed and will continue to develop quickly. Examples included in the NHS 111 triage system, the NHS Urgent Medicine Supply Advanced Service and encouraging local services. Community pharmacy will be involved in reducing visits to emergency departments and thus reducing costs. This was seen as one of the most likely developments, due to its strong presence in national policies. However, interviewees did not view community pharmacies as emergency drop-in centres, as they can only respond to a specific set of conditions.

Community pharmacies were described as the first port of call for minor illnesses, such as skin complaints and eye infections. However, some internal stakeholders believed that community pharmacies could do more with a nationally commissioned minor ailments

For peer review only - http://bmjopen.bmj.com/site/about/guidelines.xhtml

service. Independent prescribing was also a facilitator for greater involvement in this area. Providing care for minor ailments was perceived as limiting community pharmacies' ability to provide more advanced services. In addition, interviewees of all types questioned the competence of some pharmacists to provide advanced urgent care.

> *"I would expect to see definite growth…as much as NHS England talks about the prevention agenda they'll start to engage with community pharmacy which is the largest single healthcare provider of both patients and people before they get ill"*
>
> Interviewee ID14 – community pharmacist

Community pharmacies were also expected to have a prominent future role in promoting wellness and disease prevention, because of existing public health services, accessibility, well-equipped pharmacy teams and the advantage of providing care to healthy individuals. However, it was thought that community pharmacy would require improved remuneration for this role.

Subtheme: Opportunities for further community pharmacy development

> *"…pharmacies become health hubs in the future and if they did…they need to be bigger than they are today…they need to have a different look, feel, access information."*
>
> Interviewee ID9 - internal stakeholder

For peer review only

For peer review only - http://bmjopen.bmj.com/site/about/guidelines.xhtml

There was a belief that community pharmacies were well located to serve the needs of their communities and should ideally become centres for advice and health support in one location. However, some internal stakeholders suggested that pharmacies need more space, with less emphasis on retail and a more professional environment.

> *"My biggest one [hope] would be that community pharmacy would be recognised as a part of a social solution and not just a clinical solution and would they be able to sell itself…within an integrated care system…thinking about what…social value could be exploited out of community pharmacy in terms of people, place and planet."*
>
> Interviewee ID8 – external stakeholder

Interviewees envisaged a future where community pharmacy's role included tackling health inequalities, including ensuring that care was accessible in socially deprived areas. This might involve a system of 'prescriptions' for pharmacy services and involvement in social prescribing schemes.

> *"Absolutely there is opportunity for us to help people with mental health needs both from a chronic and low level to the more acute; there's definitely stuff that community pharmacy can do."*
>
> Interviewee ID5 – internal stakeholder

Mental health care in community pharmacies was presented as an opportunity, due to increasing demand and national recognition. Some community pharmacists described

For peer review only

For peer review only - http://bmjopen.bmj.com/site/about/guidelines.xhtml

mental health services that were currently provided included advice, reassurance and signposting. Community pharmacies were seen as advantageous for providing mental health care due to their convenient location, local knowledge and patient relationships that could facilitate early detection of symptoms and initial assessment. Interviewees proposed a range of interventions that might be provided, including signposting, lifestyle advice, medication adherence monitoring, adverse effect management and provision of specialist services such as Cognitive Behavioural Therapy in consultation rooms. Some strongly believed that antidepressants should be included in the NHS New Medicines Service, describing their exclusion (at the time of data collection) as *"appalling"* (ID23). However, some interviewees described mental health services as the least likely development, due to lack of resources and training needs.

Community pharmacists described the success of influenza vaccinations in community pharmacies, which are convenient and reduce GP workload. Based on this, interviewees described opportunities to expand community pharmacists' role in vaccination and administering injections including denosumab, vitamin B12, antipsychotic depot injections and corticosteroids, although others were reluctant to see this.

Subtheme: Creating the environment for future developments

> *"We are not really too concerned about the level of care that we give to our patient and again that purely goes down to the way we are funded…if we were to pay*

For peer review only - http://bmjopen.bmj.com/site/about/guidelines.xhtml

pharmacies more for hitting quality targets, for looking after patients…I think then we would see better care...”

Interviewee ID22 – community pharmacist

Interviewees could not envisage an ideal pharmacy services model without greater funding. They argued that reimbursement based on dispensing volume instead of service quality was not fit for purpose and hindered the community pharmacy workforce in using its clinical expertise to fully benefit patients. Some community pharmacists also expressed disappointment at providing clinical services without being reimbursed.

“I think one of the challenges for pharmacy is consistency and continuity…if we’ve got lots of disparate offerings from lots of different pharmacies that confuses patients, whereas what I want to see is a more standardised nationalised commissioning of services.”

Interviewee ID23 – internal stakeholder

The need for support from commissioners at all levels was acknowledged. Some community pharmacists described feeling lost due to the lack of a coherent approach to commissioning, referring to NHS England’s ‘Community Pharmacy Clinical Services Review’ as abandoned and to unfulfilled plans for newly commissioned services. They believed that long-term plans would allow investment, highlighting the influence of local commissioners and the importance of consistency.

For peer review only

For peer review only - http://bmjopen.bmj.com/site/about/guidelines.xhtml

External stakeholders from a commissioning background stated that a coherent commissioning model was one of the biggest challenges for pharmacy services, highlighting the current model's complexity. Commissioning for community pharmacy was described as fragmented compared with commissioning of other primary care providers and more integrated thinking was required.

> *"Improve our image with the community…because if you ask a patient 'well what do pharmacists do?' they just think we pop up in the back of a pharmacy, wear lab coats and hand them out…this is one of the reasons why other healthcare professionals don't even trust us as much as they should. It is because our professional body hasn't done enough to demystify our skills."*
>
> Interviewee ID22 – community pharmacist

Many community pharmacists felt dissatisfied with their representation. They referred to the lack of a *"true voice for pharmacists"* (ID14) in influential bodies, at every level of the NHS, and to other healthcare professionals and the public.

> *"… in my 13 years as a qualified pharmacist, I have not seen anything from either NHS England, the Chief Pharmaceutical Officer, the Department of Health and Social Care or Her Majesty's Government, I have seen no action that actually backs up their nice words for community pharmacy. I don't see that they trust community pharmacy to do anything other than simply dole out medicines. In fact, that is the precise term*

For peer review only

For peer review only - http://bmjopen.bmj.com/site/about/guidelines.xhtml

591 *that NHS England Chief Executive said, why are we spending all this money doling out*
592 *medicines which was really quite an offensive thing to say but that is all the NHS sees*
593 *us for."*

594 Interviewee ID24 – community pharmacist

595

596 It was important to have pharmacists working in strategic positions in clinical commissioning
597 groups (since replaced by integrated care boards) and the Department of Health and Social
598 Care to drive future changes. Support from other representatives such as the Royal
599 Pharmaceutical Society, Pharmaceutical Services Negotiating Committee and Chief
600 Pharmaceutical Officer was considered essential. Interviewees referred to inadequate
601 national support and leadership for community pharmacy. Many internal stakeholders
602 criticised the Royal Pharmaceutical Society as a representative body, stating that it did not
603 advocate beyond medicines supply and questioned whether it would be fit for purpose in
604 the future. Some external stakeholders highlighted the need for the pharmacy profession to
605 speak with one voice. All of this led to poor recognition of the sector by the state.
606 Interviewees with policymaking experience were still unaware of the role of community
607 pharmacy and highlighted the need to define the role of community pharmacy in policy.
608 There was a disconnect between the governmental and community pharmacy view of the
609 sector's strengths, with the need for the government to trust community pharmacy and see
610 it as "integral".

611

612 Evidence was considered an important factor that would contribute to better recognition
613 for community pharmacy. Interviewees from all backgrounds acknowledged the importance

For peer review only - http://bmjopen.bmj.com/site/about/guidelines.xhtml

of strengthening the evidence base for community pharmacist interventions through enhanced research and intervention recording. Creating evidence responsive to NHS challenges was significant. An external stakeholder with commissioning experience referred to the need for *"iterative"*, *"applicable at a wider scale," "affordable" and "timely"* evidence (ID17), while another identified a *"huge research gap*" (ID8) for community pharmacy interventions with an impact on social care.

Some suggested that all community pharmacy contractors work together to do this, with multiple chain pharmacies described as *"big data power houses"* (ID15). There were contrasting views regarding the influence of evidence. Most believed that pilot services, trials, local schemes and good practice could lead to change by replication at a national level. However, some community pharmacists did not share this belief.

4.0 Discussion

This study describes the views of a wide variety of systematically selected stakeholders' on how community pharmacy services may be provided in the short-to-midterm, in order to meet current NHS challenges. All types of stakeholder anticipated increased provision of services for long-term conditions, urgent care and public health, and viewed collaboration with general practice, greater use of technology, workforce development, and financial and contractual reforms as facilitators to enable this. Several barriers to these developments were also identified, which were especially apparent in the varying perspectives of internal and external stakeholders. These included an insecure and demotivated workforce, poor

For peer review only - http://bmjopen.bmj.com/site/about/guidelines.xhtml

relationships between community pharmacy and general practice, and inadequate and disunited leadership and representation.

Expectations that community pharmacists will provide services for long-term conditions, urgent care and public health have been previously reported (19) and are supported by evidence of the effectiveness of such interventions (4-6). Since the interviews were completed, a number of new nationally commissioned services in these areas have been introduced, including the Discharge Medicines Service, the Community Pharmacist Consultation Service (with GP referrals), the Smoking Cessation Service and the Hypertension Case Finding Service (20). These services demonstrate the importance of greater collaboration between community pharmacy and general practice, as some require referral of patients between these two settings, and many were initially part of the Pharmacy Integration Fund (PhIF). This was established in 2016 to accelerate the integration of pharmacy services into the wider NHS (21). The PhIF demonstrates the importance of nationally-led piloting and commissioning of pharmacy services, as this overcomes local difficulties in community pharmacy-GP relationships. More recently, the PhIF has funded a community pharmacy clinical lead in all areas of England to further integrate new pharmacy services into the wider NHS. The recent NHS England announcement of investment in community pharmacy-based services to treat common conditions, provide oral contraception and monitor blood pressure, with the aim of reducing workload in general practice, continues this trend (22).

For peer review only - http://bmjopen.bmj.com/site/about/guidelines.xhtml

However, given the importance of collaboration, actions to overcome the difficult community pharmacy-GP relations described by interviewees will be important, especially as this has been reported before (19, 23). Moreover, this study found for the first time that such competition extends to relationships between community and GP pharmacists, although other intra-professional tensions, lack of awareness and concern about the financial viability of community pharmacy have been observed previously (24, 25). The growing number of GP pharmacists means that GPs now have greater experience of the benefits of working with the pharmacy profession. This provides an opportunity for individual pharmacists and pharmacy organisations to improve local relationships with GPs, to the benefit of patients and the NHS. For example, interprofessional education is now a requirement in initial training for both pharmacists and doctors and has been shown to improve attitudes towards interprofessional collaboration (26).

Aspects of workforce development were also key facilitators identified in this study, particularly independent prescribing qualification for pharmacists and extended roles for pharmacy technicians. Although a number of recent initiatives have provided funding for independent prescribing training for community pharmacists, in 2021 there was only one community pharmacist independent prescriber for every ten community pharmacies, an unchanged proportion from 2017 (27). All newly qualified pharmacists will be independent prescribers from 2026, but further initiatives are required to train the existing workforce.

Better use of automation and technology will be required to use a more skilled workforce efficiently. The NHS currently lacks the national IT infrastructure required to give all

For peer review only - http://bmjopen.bmj.com/site/about/guidelines.xhtml

community pharmacists access to patients' full health records, which was a key facilitator of collaboration with GPs identified in this and other studies (19). However, a number of local pilot schemes are underway, with a target for national access by March 2025 (28). Interviewees also identified automated dispensing as releasing community pharmacists to provide new services. While there is evidence of the safety, efficiency and cost-effectiveness of automated community pharmacy dispensing (29), this has not yet been widely adopted by community pharmacies in England.

Financial and contractual reforms to balance income from retail and cognitive activities were identified as important facilitators of future pharmacy services (30). This is demonstrated by the rapid uptake of newly commissioned national services, such as the Community Pharmacist Consultation Service and the Hypertension Case Finding Service (over 98,000 and 113,000 consultations in October 2022, respectively) (31). The national commissioning of pharmacy services is thus a strong driver for change, particularly as it means patients have access to a consistent range of services from most community pharmacies, encouraging greater use. The PhIF model of national commissioning of services that have undergone a successful centrally funded pilot (21) should therefore be continued, to ensure consistent integration of community pharmacy with the wider NHS in all areas of England.

Such developments will only happen with strong leadership, from both the NHS and the community pharmacy sector. Both internal and external stakeholders were clear that community pharmacy leadership and representation is currently poor, particular because it

For peer review only

For peer review only - http://bmjopen.bmj.com/site/about/guidelines.xhtml

is provided by multiple organisations that do not present a unified position. Stronger, unified leadership of community pharmacy is therefore also required.

4.1 Strengths and limitations

The key strength of this study is its examination of the development of community pharmacy services in England in their entirety (rather than individual services) from the perspective of a wide variety of systematically selected interviewees, making it more representative and generalisable than some previous studies. However, the inability to recruit stakeholders from the Government and organisations that represent the public limits the breadth of perspectives included. A separate study examining the perspectives of members of the public has been completed (18). The addition of pharmacists working in other sectors (e.g. GP pharmacists) could have added useful insights. Despite the interviews being conducted in 2018-19, the results remain salient, if not more so, due to the intervening Covid-19 pandemic which has accelerated development of many of the topics discussed.

4.2 Recommendations

Based on these findings, the Department of Health and Social Care and NHS should implement policies that ensure that community pharmacy is fully integrated with the wider primary care system and can work collaboratively with other healthcare professionals (e.g. by enabling full access to health records with appropriate safeguards). Clinical and contractual incentives for the primary care sector should be aligned to avoid creating competitive relationships between professions. In addition, the state should facilitate the

For peer review only - http://bmjopen.bmj.com/site/about/guidelines.xhtml

use of automated dispensing in community pharmacy, to reduce the amount of community pharmacy staff time used for dispensing services while ensuring access to urgent medicines and face-to-face contact with a pharmacist. The time thus released should then be used to provide new services related to long-term conditions, urgent care, minor ailments and public health, but this may also include reducing health inequalities, providing vaccination and support for people with mental health problems. Commissioning of community pharmacy services should be reformed to create a less fragmented national system with adequate renumeration based on patient-orientated services rather than dispensing volume. Finally, policies are required to develop the skills of the community pharmacy workforce for both current and anticipated roles, with a particular focus on independent prescribing and pharmacy technicians.

Pharmacy representative organisations should ensure that the community pharmacy sector is represented effectively to multiple audiences (the Government, NHS, other professions and the public) and presents a unified voice and shared vision. They should especially advocate for the implementation of the policies recommended above and increase awareness of the role of community pharmacists amongst other healthcare professionals and the public. They should also promote a culture of participation in research to generate and publish an evidence-base for community pharmacy services that is responsive to NHS needs, as interviewees identified the need for a stronger evidence base for community pharmacist interventions. Finally, community pharmacy businesses and community pharmacists should be proactive in supporting these developments (as interviewees identified resistance to change as one of the biggest barriers to community pharmacy

For peer review only

For peer review only - http://bmjopen.bmj.com/site/about/guidelines.xhtml

development) and need to take urgent action to support their insecure and demotivated workforce. It should be recognised that some of these recommendations are ambitious and implementation will require careful planning and considerable time.

5.0 Conclusion

Stakeholders expect that in the future community pharmacists will help to address current NHS challenges by managing long-term conditions, providing urgent care and public health services. Collaboration with primary care and use of new technologies will be fundamental for facilitating these changes and ensuring they benefit patients. Numerous changes are required from the Government, NHS, pharmacy representative bodies, community pharmacy businesses and community pharmacists to create the environment in which these changes can occur. Future research should assess the impact of these changes (especially the effectiveness of new services) and understand more about the evolving professional relationships between community pharmacists, GPs and general practice pharmacists.

Author contributions

EP: Conceptualization; Methodology; Project administration; Data curation; Formal analysis; Writing – original draft; Writing – original draft.

PO: Conceptualization; Methodology; Supervision; Writing - review & editing.

MW: Conceptualization; Methodology; Supervision; Funding acquisition; Writing - review & editing.

For peer review only - http://bmjopen.bmj.com/site/about/guidelines.xhtml

MDJ: Conceptualization; Methodology; Supervision; Funding acquisition; Writing – review & editing.

Acknowledgements

The authors would like to sincerely thank all the stakeholders who participated in the study and everyone who helped with their recruitment. The authors would also like to thank Dr Bharat Shah CBE, Sigma Pharmaceuticals and the Harold and Marjorie Moss Charitable Trust Fund. Dr Paloumpi's PhD was funded by The Bharat Shah PhD Scholarship. Dr Paloumpi was also supported by the Harold and Marjorie Moss Charitable Trust Fund.

References

1. Razai MS and Majeed A. General practice in England: the current crisis, opportunities, and challenges. *J Ambul Care Manag* 2022; 45: 135-139. DOI: 10.1097/JAC.0000000000000410.
2. Cooksley T, Clarke S, Dean J, et al. NHS crisis: rebuilding the NHS needs urgent action. *BMJ* 2023; 380: 1. 20230103. DOI: 10.1136/bmj.p1.
3. Baird B and Beech J. Community pharmacy explained https://www.kingsfund.org.uk/publications/community-pharmacy-explained (2020, accessed 20 January 2021).
4. Evans CD, Watson E, Eurich DT, et al. Diabetes and cardiovascular disease interventions by community pharmacists: A systematic review. *Ann Pharmacother* 2011; 45: 615-628. DOI: 10.1345/aph.1P615.
5. Watson M, Holland R, Ferguson J, et al. *Community pharmacy management of minor illness (MINA study)*. 2014. London, UK: Pharmacy Research UK.
6. Thomson K, Hillier-Brown F, Walton N, et al. The effects of community pharmacy-delivered public health interventions on population health and health inequalities: A review of reviews. *Prev Med* 2019; 124: 98-109. DOI: 10.1016/j.ypmed.2019.04.003.
7. Royal College of Emergency Medicine, Royal College of General Practitioners, Royal College of Physicians, et al. Rebuilding the NHS: better medical pathways for acute care 2022, https://www.rcplondon.ac.uk/projects/outputs/rebuilding-nhs-better-medical-pathways-acute-care-2022 (2022, accessed 31st January 2023).
8. Ernst and Young. *Impacts of current funding, policy and economic environment on independent pharmacy in England* 2020. London, UK.
9. Wickware C and Wilkinson E. The new abnormal: vulnerable patients hit by pharmacy closures. *Pharm J* 2020; 305. DOI: 10.1211/PJ.2020.20208448.
10. Wickware C. Online pharmacy dispensing volume grows by 45% in 2020, fuelled by COVID-19 pandemic. *Pharm J* 2021; 306. DOI: 10.1211/PJ.2021.1.78119.

For peer review only - http://bmjopen.bmj.com/site/about/guidelines.xhtml

11. Alshehri AA, Cheema E, Yahyouche A, et al. Evaluating the role and integration of general practice pharmacists in England: a cross-sectional study. *Int J Clin Pharm* 2021; 43: 1609-1618. 20210602. DOI: 10.1007/s11096-021-01291-6.
12. Wright D, Twigg M, Family H, et al. *Independent review of community pharmacy contractor representation and support: "Providing best value for contractors"*. 2020. Norwich, UK.
13. Varvasovszky Z and Brugha R. How to do (or not to do) a stakeholder analysis. *Health Policy Plan* 2000; 15: 338–345.
14. Paloumpi E, Ozieranski P, Watson MC, et al. Professional and governmental policy on community pharmacy: a 10-year policy review and comparative analysis (2008-2017). *Explor Res Clin Soc Pharm* 2023; 11: 100298. DOI: 10.1016/j.rcsop.2023.100298.
15. Harvey WS. Strategies for conducting elite interviews. *Qual Res* 2011; 11: 431-441. DOI: 10.1177/1468794111404329.
16. Walt G and Gilson L. Reforming the health sector in developing countries: the central role of policy analysis. *Health Policy Plan* 1994; 9: 353-370. DOI: 10.1093/heapol/9.4.353.
17. Braun V and Clarke V. Using thematic analysis in psychology. *Qual Res Psychol* 2006; 3: 77-101. DOI: 10.1191/1478088706qp063oa.
18. Paloumpi E, Ozieranski P, Watson MC, et al. Pharmacy users' perceptions, awareness and future expectations of community pharmacy in England: a focus group study. *Int J Pharm Prac* 2023; Revision submitted.
19. Hindi AMK, Schafheutle EI and Jacobs S. Community pharmacy integration within the primary care pathway for people with long-term conditions: a focus group study of patients', pharmacists' and GPs' experiences and expectations. *BMC Fam Pract* 2019; 20: 26. DOI: 10.1186/s12875-019-0912-0.
20. Pharmaceutical Services Negotiating Committee. National pharmacy services, https://psnc.org.uk/national-pharmacy-services/ (2022, accessed 31st January 2023).
21. NHS England. Pharmacy Integration Programme, https://www.england.nhs.uk/primary-care/pharmacy/pharmacy-integration-fund/ (2023, accessed 1st February 2023).
22. NHS England. *Delivery plan for recovering access to primary care*. 9th May 2023. London: NHS England.
23. Hindi AMK, Jacobs S and Schafheutle EI. Solidarity or dissonance? A systematic review of pharmacist and GP views on community pharmacy services in the UK. *Health Soc Care Community* 2019; 27: 565-598. DOI: 10.1111/hsc.12618.
24. Karampatakis GD, Patel N, Stretch G, et al. Community pharmacy teams' experiences of general practice-based pharmacists: an exploratory qualitative study. *BMC Health Serv Res* 2020; 20: 431. DOI: 10.1186/s12913-020-05245-y.
25. Nabhani-Gebara S, Fletcher S, Shamim A, et al. General practice pharmacists in England: Integration, mediation and professional dynamics. *Res Social Adm Pharm* 2020; 16: 17-24. 20190201. DOI: 10.1016/j.sapharm.2019.01.014.
26. Lapkin S, Levett-Jones T and Gilligan C. A systematic review of the effectiveness of interprofessional education in health professional programs. *Nurse Educ Today* 2013; 33: 90-102. 20111222. DOI: 10.1016/j.nedt.2011.11.006.
27. Centre for Pharmacy Workforce Studies. *Community pharmacy workforce survey 2021*. 2022. Health Education England.
28. Wickware C. Patient records: when will community pharmacies get access? *Pharm J* 2022; 309. DOI: 10.1211/PJ.2022.1.153943.
29. Sng Y, Ong CK and Lai YF. Approaches to outpatient pharmacy automation: a systematic review. *Eur J Hosp Pharm* 2019; 26: 157-162. DOI: 10.1136/ejhpharm-2017-001424.
30. Mossialos E, Courtin E, Naci H, et al. From "retailers" to health care providers: Transforming the role of community pharmacists in chronic disease management. *Health Policy* 2015; 119: 628-639. DOI: 10.1016/j.healthpol.2015.02.007.

For peer review only - http://bmjopen.bmj.com/site/about/guidelines.xhtml

31. PharmData Ltd. PharmData, https://www.pharmdata.co.uk/ (2023, accessed 1st February 2023).

For peer review only

For peer review only - http://bmjopen.bmj.com/site/about/guidelines.xhtml

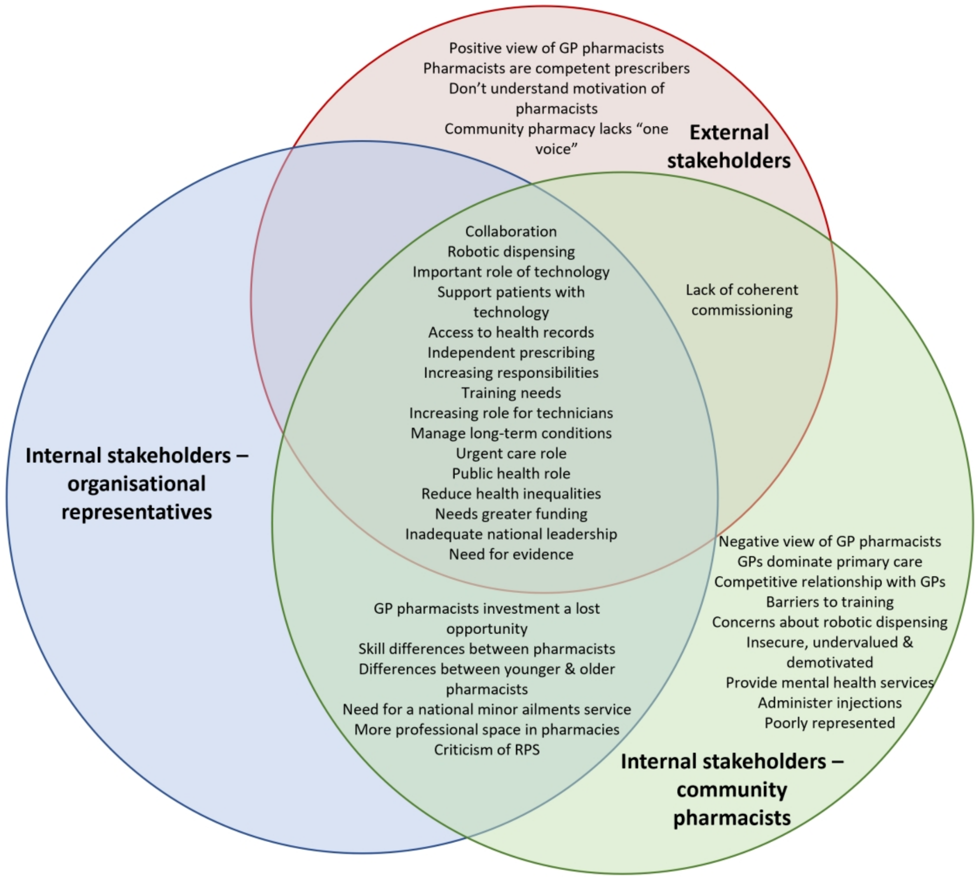

Figure 1: Areas of convergence and divergence in the views discussed by external stakeholders and internal stakeholders (both organisational representatives and community pharmacists).

532x470mm (130 x 130 DPI)

BMJ Open

Professional stakeholders' expectations for the future of community pharmacy practice in England: a qualitative study

Supplemental material

Evgenia Paloumpi[a]*, Piotr Ozieranski[b], Margaret C Watson[c] and Matthew D. Jones[a]

[a]Department of Life Sciences, University of Bath, Bath, England.

[b]Department of Social & Policy Sciences, University of Bath, Bath, England.

[c]Strathclyde Institute of Pharmacy and Biomedical Sciences, University of Strathclyde, Glasgow, Scotland.

*The views expressed in this paper are purely those of the author. They do not necessarily reflect the views or official positions of the European Commission and the ERC Executive Agency.

For peer review only

Table S1: Consolidated criteria for reporting qualitative research (COREQ) 32-item checklist

	Guide questions/description	Comment	Location in the manuscript (section)
**Domain 1: Research team and reflexivity**			
Personal Characteristics			
1. Interviewer/ facilitator	Which author/s conducted the interview or focus group?	Evgenia Paloumpi (EP) conducted the interviews.	2.2
2. Credentials	What were the researcher's credentials? E.g. PhD, MD	EP had MPharm and MRes degrees. MW, PO and MJ each had a PhD. EP, MW and MJ were all registered pharmacists.	N/A
3. Occupation	What was their occupation at the time of the study?	EP was a PhD student at Department of Pharmacy and Pharmacology, University of Bath.<br>MW, PO and MJ were EP's supervisors and full-time academics.	N/A
4. Gender	Was the researcher male or female?	EP and MW were female.<br>PO and MJ were male.	N/A
5. Experience and training	What experience or training did the researcher have?	EP had undertaken several courses related to advanced qualitative health research before the study was conducted. The other authors had extensive training and experience in qualitative health research.	N/A
Relationship with participants			
6. Relationship established	Was a relationship established prior to study commencement?	EP had previously met some participants at professional events such as conferences.	N/A
7. Participant knowledge of the interviewer	What did the participants know about the researcher? e.g. personal goals, reasons for doing the research	Potential participants were sent written information explaining the aims of the study and that it informed part of a PhD project.<br><br>Prior the interviews, EP introduced herself again as a PhD student and described the purpose of the project, before answering any questions that participants may have had.	2.1

8. Interviewer characteristics	What characteristics were reported about the interviewer/facilitator? e.g. Bias, assumptions, reasons and interests in the research topic	EP is a pharmacist with a particular interest in health policy.<br>MW is a pharmacist with a particular interest in community pharmacy research.<br>MJ is a pharmacist with experience of work as a community pharmacy locum and research interests in medicines safety and medicines information.<br>PO is a health policy researcher.	N/A
**Domain 2: Study design**			
Theoretical framework			
9. Methodological orientation and Theory	What methodological orientation was stated to underpin the study? e.g. grounded theory, discourse analysis, ethnography, phenomenology, content analysis	Underpinning ontology and epistemology were interpretivist. A reflexive thematic analysis using inductive approaches was applied. The Walt and Gilson policy framework was used to develop the topic guide.	2.3
Participant selection			
10. Sampling	How were participants selected? e.g. purposive, convenience, consecutive, snowball	Organisational representatives were selected using stakeholder mapping and snowballing. Community pharmacists were selected purposively.	2.1
11. Method of approach	How were participants approached? e.g. face-to-face, telephone, mail, email	Organisational representatives were contacted directly in writing and by telephone.<br><br>Community pharmacists were approached via advertisements in the Royal Pharmaceutical Society online community pharmacy forum, 48 local pharmaceutical committees, the researchers' networks and social media.<br><br>A study information sheet was emailed to these individuals who agreed to take part.	2.1
12. Sample size	How many participants were in the study?	Twenty-five	3.1

For peer review only

13. Nonparticipation	How many people refused to participate or dropped out? Reasons?	No interviewees were recruited from the following groups: public representatives; pharmacy and allied healthcare professionals (The Association of Independent Multiple Pharmacies, Boots Pharmacists Association, Royal College of General Practitioners, British Medical Association, Royal College of Nursing); commissioners and policymakers (Department of Health and Social Care, All-Party Pharmacy Group); and experts and collaborators (The King's Fund). The most common reasons for non-recruitment were either no response received or time restrictions of interviewees.	N/A
Setting			
14. Setting of data collection	Where was the data collected? e.g. home, clinic, workplace	Interviewees could determine whether they preferred to be interviewed face-to-face or by telephone. All face-to-face interviews took place in nonclinical professional area.<br><br>Five interviews were conducted in person at a location convenient to participants (e.g. participants' offices), 19 by telephone and one by Skype using videocall.	2.2
15. Presence of non-participants	Was anyone else present besides the participants and researchers?	No.	N/A
16. Description of sample	What are the important characteristics of the sample? e.g. demographic data, date	-	3.1 and Table S2 in this supplementary material
Data collection			
17. Interview guide	Were questions, prompts, guides provided by the authors? Was it pilot tested?	An interview topic guide (see below) was developed based on a recent policy review, previous research, supervisory team discussions and the Walt and Gilson policy framework (content, context, process and actors). The final interview guide included 11, broad, open-ended questions and probes to allow a flexible conversation with participants, with the aim of understanding their views on how	Below

For peer review only

		pharmaceutical care in the community would be provided in 2030. The topic guide and interviewer's technique were piloted interview with a stakeholder who was not included in the study as a participant. The pilot provided useful insights and feedback on interview conduct (e.g. ensuring availability for the interview duration before commencing it). The topic guide evolved progressively as interviews were carried out, to ensure that findings informed subsequent interviews. Questions 7, 8 and 9 were added to the topic guide as many participants referred to these topics during their interviews.	
18. Repeat interviews	Were repeat interviews carried out? If yes, how many?	No.	N/A
19. Audio/visual recording	Did the research use audio or visual recording to collect the data?	All interviews were audio-recorded (with permission) and later transcribed verbatim.	2.2
20. Field notes	Were field notes made during and/or after the interview or focus group?	Hand-written notes with key points and observations of the interview were also made by the researcher.	2.2
21. Duration	What was the duration of the interviews or focus group?	Interview duration varied from 35 to 75 minutes (mean = 60 minutes).	3.1
22. Data saturation	Was data saturation discussed?	Participants in this study were not recruited based on data saturation estimates as every stakeholder's view was considered useful for the future development of pharmaceutical care. Recruitment continued until all identified stakeholders had been interviewed or had declined.	2.1
23. Transcripts returned	Were transcripts returned to participants for comment and/or correction?	Transcripts were not returned to participants for comments due to time restrictions.	N/A
**Domain 3: Analysis and findings**			
Data analysis			

For peer review only

24. Number of data coders	How many data coders coded the data?	Coding was led by one researcher (EP), with regular discussion and agreement with the team.	2.3
25. Description of the coding tree	Did authors provide a description of the coding tree?		3.2-2.6
26. Derivation of themes	Were themes identified in advance or derived from the data?	This study used an inductive approach. The codes and the themes were informed by interview content.	2.3
27. Software	What software, if applicable, was used to manage the data?	NVivo12® software was used.	2.3
28. Participant checking	Did participants provide feedback on the findings?	No.	N/A
Reporting			
29. Quotations presented	Were participant quotations presented to illustrate the themes/findings? Was each quotation identified? e.g. participant number	Yes, quotations were presented and identified in a manner protecting participant confidentiality.	3.3-3.6
30. Data and findings consistent	Was there consistency between the data presented and the findings?	Yes, there was consistency between the data and the findings.	3.2-3.6
31. Clarity of major themes	Were major themes clearly presented in the findings?	Yes, major themes were clearly identified.	3.2-3.6
32. Clarity of minor themes	Is there a description of diverse cases or discussion of minor themes?	Yes, minor themes and diverse cases were clearly identified and related to major themes.	3.2-3.6

N/A; not applicable

Tong A, Sainsbury P, and Craig J. Consolidated criteria for reporting qualitative research (COREQ): a 32-item checklist for interviews and focus groups. *Int J Qual Health Care* 2007;19:349–57.

For peer review only

Table S2: Organisations with representatives invited to participate in this study

Type of stakeholder	Type of organisation	Organisations
External stakeholders	Public representatives	Healthwatch<br>National Voices<br>The Patients' Association
	Health professions	Royal College of General Practitioners (RCGP)<br>British Medical Association (BMA)<br>Royal College of Nursing (RCN)
	Commissioners and policymakers	Department of Health and Social Care (DHSC)<br>All Party Parliamentary Group on Pharmacy (APPG)<br>NHS England (NHSE)<br>NHS Clinical Commissioning (NHSCC)
	Experts and collaborators	UK universities<br>The King's Fund<br>Pharmacy media
Internal stakeholders	Pharmacy organisations	Royal Pharmaceutical Society (RPS)<br>Pharmaceutical Services Negotiating Committee (PSNC)<br>National Pharmacy Association (NPA)<br>Company Chemists' Association (CCA)<br>The Association of Independent Multiple Pharmacies (AIMp)<br>Pharmacists' Defence Association (PDA)<br>Boots Pharmacists' Association (BPA)<br>Association of Pharmacy Technicians UK (APTUK)

For peer review only

Interview topic guide

Opening

- Researcher introduction and brief overview explaining purpose of the study and describing the term "pharmaceutical care in the community" used for the study

  "My name is Evina and I am a PhD student from University of Bath. As you already know, my research aims to develop a more evidence-based vision for the future of community pharmacy services in England. We are having this conversation to discuss your views on how pharmaceutical care will be provided in the community in the year 2030. And when I say pharmaceutical care, I would describe this discipline as helping patients use and manage medicines effectively, but also providing wider interventions that could create an impact on their quality of life."

- Check participant has read Participant Information Sheet (provide a copy and time to read if they haven't)
- Ask participant if they have any questions about the study
- Explain to the participants about the informed consent
- Ask how much time the participant has available and plan questions – "*Our interview is not expected to last more than one hour but if you have other commitments, we can finish earlier than that."*
- Secure consent
- Start recording the interview

Main Discussion

Probes will be used flexibly, in response to participant's answers.

1. How would you describe your [members'] role in relation to this sort of pharmaceutical care in the community?	**Role**:
2. In your view, in what ways might pharmaceutical care in the community be provided in the year 2030?<br><br>*Probe common topics from policy review:*<br>• *urgent and emergency care*<br>• *mental health (dementia friend)*<br>• *closer collaborations with primary care professionals*<br>• *long-term conditions*<br>• *patient-centred care*<br>• *new models of care*	**Ways**:
3. Which of these developments are more like likely to happen and why?	**More likely to happen & why**:

For peer review only

Probe common drivers from policy review:<br>• *unnecessary workload in general practice*<br>• *increased service demands*<br>• *funding allocation*<br>• *increased hospital admissions*	
4. We have already discussed the different ways you think that pharmaceutical care will be provided in the year 2030. Suppose that you had an opportunity to create the ideal model of pharmaceutical care in the community, but without increasing the total cost. What would this ideal model include?	**Ideal model:**
5. [If answer to Q4 different to Q3] Your ideal model is different to what you think will actually happen. Why do you think your ideal model is unlikely to come about?	**Why expectation different to ideal:**
6. What would make your ideal model more likely to come about?<br><br>*Probe common facilitators and barriers for Q5 and Q6:*<br>• *individual behaviour*<br>• *utilising technology*<br>• *supporting workforce*<br>• *providing evidence*<br>• *facilitate commissioning*	**What would make ideal more likely:**
7. What are the current barriers to pharmaceutical care provision in the community?	**Barriers:**
8. What are your views on the role of pharmacy technicians in pharmaceutical care?	**Technicians:**
9. What are your views on community pharmacy mergers and closures due to financial constraints?	**Mergers and closures:**
10. Do you feel there is anything else you would like to add?	**Additional:**

For peer review only

Closing

- Summarise main points of the discussion
- One last question from me "What other organisation or individual would you consider influential for the future of pharmaceutical care in the community?
- Ask if they would like a copy of the results to be sent to them
- Thank participants for their time

For peer review only

Table S3: Interviewee characteristics

Type of interviewee	Number of interviewees	Organisations with whom interviewees were linked
**External stakeholders**	7<br>(4 female, 3 male)	NHS England<br>NHS Clinical Commissioning<br>General practice<br>National Association of Primary Care<br>New NHS Alliance<br>UK universities<br>Pharmacy-related media
**Internal stakeholders – organisational representatives**	8<br>(5 female, 3 male)	Royal Pharmaceutical Society<br>Pharmaceutical Services Negotiating Committee<br>National Pharmacy Association<br>Company Chemists' Association<br>Pharmacists' Defence Association[1]<br>Association of Pharmacy Technicians UK<br>Pharmaceutical industry
**Internal stakeholders – community pharmacists[2,3]**	10<br>(2 female, 8 male)	Independent community pharmacy (n=7)<br>Multiple community pharmacy (n=7)<br>Locum pharmacist (n=4)<br>Local pharmaceutical committees (n=2)<br>Pharmacy education bodies (n=2)

[1]Two interviewees linked to the Pharmacists' Defence Association were interviewed

[2]A multiple community pharmacy contractor was defined as consisting of six pharmacies or more; contractors with five or fewer pharmacies were regarded as independent.

[3]Some community pharmacists were linked to more than one type of organisation.

For peer review only

Table S4: Characteristics of interviewees from all backgrounds

ID number	Organisational stakeholders			Community pharmacist internal stakeholders				
	External	Internal	Gender	Independent community pharmacy[1]	Multiple community pharmacy[1]	Locum	Gender	Region in England
**1**				✓	✓	✓	Male	North
**2**		✓	Male					
**3**		✓	Female					
**4**		✓	Male					
**5**		✓	Male					
**6**				✓	✓	✓	Female	Midlands and East
**7**				✓			Male	North
**8**	✓		Female					
**9**		✓	Female					
**10**				✓			Male	Midlands and East
**11**		✓	Female					
**12**	✓		Male					
**13**				✓	✓		Male	North
**14**				✓			Male	South
**15**	✓		Male					
**16**	✓		Female					
**17**	✓		Female					
**18**		✓	Female					
**19**					✓		Male	South
**20**	✓		Male					
**21**					✓	✓	Female	South
**22**				✓	✓	✓	Male	North, Midlands and East
**23**		✓	Female					
**24**					✓		Male	North
**25**	✓		Female					
**TOTAL**	**7**	**8**	**Male (n=6) Female (n=9)**	**7**	**7**	**4**	**Male (n=8) Female (n=2)**	-

[1]A multiple community pharmacy contractor was defined as consisting of 6 pharmacies or more; contractors with 5 or fewer pharmacies were regarded as independent (General Pharmaceutical Services in England 2008/09 - 2018/19, NHS Digital).

For peer review only

	Guide questions/description	Comment	Location in the manuscript (section)
**Domain 1: Research team and reflexivity**			
Personal Characteristics			
1. Interviewer/ facilitator	Which author/s conducted the interview or focus group?	Evgenia Paloumpi (EP) conducted the interviews.	2.2
2. Credentials	What were the researcher's credentials? E.g. PhD, MD	EP had MPharm and MRes degrees. MW, PO and MJ each had a PhD. EP, MW and MJ were all registered pharmacists.	N/A
3. Occupation	What was their occupation at the time of the study?	EP was a PhD student at Department of Pharmacy and Pharmacology, University of Bath.<br>MW, PO and MJ were EP's supervisors and full-time academics.	N/A
4. Gender	Was the researcher male or female?	EP and MW were female.<br>PO and MJ were male.	N/A
5. Experience and training	What experience or training did the researcher have?	EP had undertaken several courses related to advanced qualitative health research before the study was conducted. The other authors had extensive training and experience in qualitative health research.	N/A
Relationship with participants			
6. Relationship established	Was a relationship established prior to study commencement?	EP had previously met some participants at professional events such as conferences.	N/A
7. Participant knowledge of the interviewer	What did the participants know about the researcher? e.g. personal goals, reasons for doing the research	Potential participants were sent written information explaining the aims of the study and that it informed part of a PhD project.<br><br>Prior the interviews, EP introduced herself again as a PhD student and described the purpose of the project, before answering any questions that participants may have had.	2.1

For peer review only

8. Interviewer characteristics	What characteristics were reported about the interviewer/facilitator? e.g. Bias, assumptions, reasons and interests in the research topic	EP is a pharmacist with a particular interest in health policy.<br>MW is a pharmacist with a particular interest in community pharmacy research.<br>MJ is a pharmacist with experience of work as a community pharmacy locum and research interests in medicines safety and medicines information.<br>PO is a health policy researcher.	N/A
**Domain 2: Study design**			
Theoretical framework			
9. Methodological orientation and Theory	What methodological orientation was stated to underpin the study? e.g. grounded theory, discourse analysis, ethnography, phenomenology, content analysis	Underpinning ontology and epistemology were interpretivist. A reflexive thematic analysis using inductive approaches was applied. The Walt and Gilson policy framework was used to develop the topic guide.	2.3
Participant selection			
10. Sampling	How were participants selected? e.g. purposive, convenience, consecutive, snowball	Organisational representatives were selected using stakeholder mapping and snowballing. Community pharmacists were selected purposively.	2.1
11. Method of approach	How were participants approached? e.g. face-to-face, telephone, mail, email	Organisational representatives were contacted directly in writing and by telephone.<br><br>Community pharmacists were approached via advertisements in the Royal Pharmaceutical Society online community pharmacy forum, 48 local pharmaceutical committees, the researchers' networks and social media.<br><br>A study information sheet was emailed to these individuals who agreed to take part.	2.1
12. Sample size	How many participants were in the study?	Twenty-five	3.1

For peer review only

13. Nonparticipation	How many people refused to participate or dropped out? Reasons?	No interviewees were recruited from the following groups: public representatives; pharmacy and allied healthcare professionals (The Association of Independent Multiple Pharmacies, Boots Pharmacists Association, Royal College of General Practitioners, British Medical Association, Royal College of Nursing); commissioners and policymakers (Department of Health and Social Care, All-Party Pharmacy Group); and experts and collaborators (The King's Fund). The most common reasons for non-recruitment were either no response received or time restrictions of interviewees.	N/A
Setting			
14. Setting of data collection	Where was the data collected? e.g. home, clinic, workplace	Interviewees could determine whether they preferred to be interviewed face-to-face or by telephone. All face-to-face interviews took place in nonclinical professional area.<br><br>Five interviews were conducted in person at a location convenient to participants (e.g. participants' offices), 19 by telephone and one by Skype using videocall.	2.2
15. Presence of non-participants	Was anyone else present besides the participants and researchers?	No.	N/A
16. Description of sample	What are the important characteristics of the sample? e.g. demographic data, date	-	3.1 and Table S2 in this supplementary material
Data collection			
17. Interview guide	Were questions, prompts, guides provided by the authors? Was it pilot tested?	An interview topic guide (see below) was developed based on a recent policy review, previous research, supervisory team discussions and the Walt and Gilson policy framework (content, context, process and actors). The final interview guide included 11, broad, open-ended questions and probes to allow a flexible conversation with participants, with the aim of understanding their views on how	Below

For peer review only

		pharmaceutical care in the community would be provided in 2030. The topic guide and interviewer's technique were piloted interview with a stakeholder who was not included in the study as a participant. The pilot provided useful insights and feedback on interview conduct (e.g. ensuring availability for the interview duration before commencing it). The topic guide evolved progressively as interviews were carried out, to ensure that findings informed subsequent interviews. Questions 7, 8 and 9 were added to the topic guide as many participants referred to these topics during their interviews.	
18. Repeat interviews	Were repeat interviews carried out? If yes, how many?	No.	N/A
19. Audio/visual recording	Did the research use audio or visual recording to collect the data?	All interviews were audio-recorded (with permission) and later transcribed verbatim.	2.2
20. Field notes	Were field notes made during and/or after the interview or focus group?	Hand-written notes with key points and observations of the interview were also made by the researcher.	2.2
21. Duration	What was the duration of the interviews or focus group?	Interview duration varied from 35 to 75 minutes (mean = 60 minutes).	3.1
22. Data saturation	Was data saturation discussed?	Participants in this study were not recruited based on data saturation estimates as every stakeholder's view was considered useful for the future development of pharmaceutical care. Recruitment continued until all identified stakeholders had been interviewed or had declined.	2.1
23. Transcripts returned	Were transcripts returned to participants for comment and/or correction?	Transcripts were not returned to participants for comments due to time restrictions.	N/A
**Domain 3: Analysis and findings**			
Data analysis			

For peer review only

24. Number of data coders	How many data coders coded the data?	Coding was led by one researcher (EP), with regular discussion and agreement with the team.	2.3
25. Description of the coding tree	Did authors provide a description of the coding tree?		3.2-2.6
26. Derivation of themes	Were themes identified in advance or derived from the data?	This study used an inductive approach. The codes and the themes were informed by interview content.	2.3
27. Software	What software, if applicable, was used to manage the data?	NVivo12® software was used.	2.3
28. Participant checking	Did participants provide feedback on the findings?	No.	N/A
Reporting			
29. Quotations presented	Were participant quotations presented to illustrate the themes/findings? Was each quotation identified? e.g. participant number	Yes, quotations were presented and identified in a manner protecting participant confidentiality.	3.3-3.6
30. Data and findings consistent	Was there consistency between the data presented and the findings?	Yes, there was consistency between the data and the findings.	3.2-3.6
31. Clarity of major themes	Were major themes clearly presented in the findings?	Yes, major themes were clearly identified.	3.2-3.6
32. Clarity of minor themes	Is there a description of diverse cases or discussion of minor themes?	Yes, minor themes and diverse cases were clearly identified and related to major themes.	3.2-3.6

N/A; not applicable

Tong A, Sainsbury P, and Craig J. Consolidated criteria for reporting qualitative research (COREQ): a 32-item checklist for interviews and focus groups. *Int J Qual Health Care* 2007;19:349–57.

For peer review only
